# The unique risk factor profile of triple-negative breast cancer: a comprehensive meta-analysis

**DOI:** 10.1093/jnci/djae056

**Published:** 2024-03-05

**Authors:** Nitya Kumar, Sarah Ehsan, Shahana Banerjee, Claudia Fernandez Perez, Isabelle Lhuilier, Jillian Neuner, Tara Friebel-Klingner, Oluwadamilola M Fayanju, Bindhu Nair, Sara Anjum Niinuma, Shivangi Nampoothiri, Anne Marie McCarthy

**Affiliations:** Department of Medicine, Royal College of Surgeons in Ireland - Bahrain, Busaiteen, Bahrain; Department of Biostatistics, Epidemiology & Informatics, Perelman School of Medicine, University of Pennsylvania, Philadelphia, PA, USA; Department of Biostatistics, Epidemiology & Informatics, Perelman School of Medicine, University of Pennsylvania, Philadelphia, PA, USA; Department of Biostatistics, Epidemiology & Informatics, Perelman School of Medicine, University of Pennsylvania, Philadelphia, PA, USA; Department of Biostatistics, Epidemiology & Informatics, Perelman School of Medicine, University of Pennsylvania, Philadelphia, PA, USA; Department of Biostatistics, Epidemiology & Informatics, Perelman School of Medicine, University of Pennsylvania, Philadelphia, PA, USA; Department of Epidemiology, Bloomberg School of Public Health, Johns Hopkins University, Baltimore, MD, USA; Department of Biostatistics, Epidemiology & Informatics, Perelman School of Medicine, University of Pennsylvania, Philadelphia, PA, USA; Department of Medicine, Royal College of Surgeons in Ireland - Bahrain, Busaiteen, Bahrain; Department of Medicine, Royal College of Surgeons in Ireland - Bahrain, Busaiteen, Bahrain; Science Department, Christ University, Bangalore, KA, India; Department of Biostatistics, Epidemiology & Informatics, Perelman School of Medicine, University of Pennsylvania, Philadelphia, PA, USA

## Abstract

**Background:**

Triple-negative breast cancer (TNBC) has a poor prognosis compared with other breast cancer subtypes. This systematic review and meta-analysis examines whether known risk factors for breast cancer are also associated with TNBC in adult women.

**Methods:**

EMBASE, Medline, SCOPUS, and gray literature were queried with no limit on the date or language of publication. The exposures of interest included parity, breastfeeding, duration of breastfeeding, age at menarche, age at first live birth, oral contraceptive (OC) use, duration of OC use, use of menopausal hormone therapy (MHT), family history, body mass index (BMI), alcohol use, smoking, and breast density. The main outcome of interest was TNBC. Study quality was determined using the Newcastle-Ottawa scale for case control studies and cohort studies. We estimated weighted odds ratios from random effects models to study the exposure–outcome associations. Protocol was registered under the number: *PROSPERO 2021 CRD42021254594.*

**Results:**

Thirty-three studies were included. Family history, longer duration of OC use, and higher breast density were significantly associated with increased risk for TNBC, whereas later age at menarche, later age at first birth, and breastfeeding were protective against TNBC. Parity, MHT, alcohol, smoking, and BMI were not significantly associated with TNBC overall, but higher parity was associated with higher risk among Black women.

**Conclusion:**

Our findings highlight that TNBC has a distinct risk factor profile compared with overall breast cancer. This can be the foundational work in identification of actionable TNBC risk factors to improve prevention and early detection of these poor prognosis breast tumors.

Breast cancer is a heterogeneous disease with distinct molecular subtypes (1). The basal-like subtype, which is marked by expression of genes usually found in basal cells of the normal breast, is associated with poor prognosis (2). Immunohistochemistry is typically used to measure expression of estrogen receptor (ER), progesterone receptor (PR), and human epidermal growth factor receptor 2 (HER2) as a proxy for molecular subtype. Cancers that do not express ER, PR, or HER2 are known as triple-negative breast cancers (TNBC), which to a large extent overlap with the basal-like molecular subtype. TNBCs are more aggressive, are less likely to be detected by mammography screening than other breast cancers (3), and do not respond to therapies targeting the estrogen or HER2 pathways, making TNBCs more deadly than hormone receptor positive subtypes (4). The 5-year survival for TNBC is just over 75%, compared with 95% for ER/PR+HER2- tumors (5,6). Additionally, TNBCs have been found to have a higher prevalence among African ancestry populations (7). Given the stark differences in prognosis between subtypes, it is crucial to understand the unique risk profile for TNBC in order to aid prevention and screening efforts.

Prior studies have shown that breast cancer subtypes have unique etiologies (8). A systematic review found that most established breast cancer risk factors were associated with ER/PR+HER2- breast cancer; associations with TNBC were less consistent, partly because of fewer studies and smaller samples sizes among TNBCs (8). However, there were too few studies at the time to perform a meta-analysis. Few meta-analyses have been conducted that solely evaluate the risk factors for TNBC. Prior meta-analyses mainly have assessed reproductive and lifestyle risk factors (9-13). Only one meta-analysis, to our knowledge, has reviewed the association of breast density with ER, PR, and HER2 status; however, receptor status was analyzed individually rather than grouped into subtypes (14).

This systematic review and meta-analysis examined the associations of known breast cancer risk factors, including age, age at menarche, age at first live birth, parity, breastfeeding and breastfeeding duration, family history of breast cancer, oral contraceptive (OC) use and duration of use, age at menopause, use of menopausal hormone therapy (MHT), breast density, prior biopsy of benign breast disease, body mass index (BMI), and alcohol consumption with TNBCs in adult women.

## Methods

Our study methodology was developed a priori and was registered at PROSPERO register for systematic review protocols: registration number PROSPERO 2021 CRD42021254594 (15). All methods and findings have been reported as per PRISMA guidelines; the PRISMA checklist is present in Supplementary Table 1 (available online).

### Search strategy

Keywords that included alternative terms for exposures and outcome were drafted and reviewed by all members of the team. The search queries for specific databases were drafted by BN and reviewed by the team. We executed searches on Medline, EMBASE, and SCOPUS with no restriction on date or language of publication. A gray literature search was conducted on Google Scholar and reference lists of included studies. The detailed search queries used for each database are provided in Supplementary Figure 1 (available online). The search was initially conducted on May 26, 2021, and was repeated on May 29, 2022, and November 22, 2022.

### Study selection

Studies were screened using the web-based application Rayyan (16). Four reviewers performed the screening (NK, SE, SB, and CF) in a blinded fashion. Each reviewer checked 20% of each reviewer’s work. All conflicts pertaining to decision on exclusion/inclusion of studies were resolved in a group discussion and voting. Screening was performed in 3 stages: 1) titles + abstract screen, 2) full-text screen, and 3) screening during data extraction stage where studies were excluded if data were not available from the authors.

### Study inclusion and exclusion criteria

The outcome of interest was TNBC, and it was defined as invasive breast cancer tumors that are ER-, PR-, and HER2- as assessed by immunohistochemistry, fluorescent in situ hybridization, or genomic profiling. Studies based on biological males or participants with unilateral or bilateral mastectomy were excluded. To ensure minimum study quality and assess temporality, we restricted to cohort and case-control designs. Studies were included if they compared occurrence of TNBC with no cancer and excluded if the comparison group were not cancer-free.

### Exposures

All studies that reported association between TNBC and any of the following exposures were included: age, age at menarche, age at first live birth, parity, breastfeeding, duration of breastfeeding, family history of breast cancer, OC use, duration of OC use, use of MHT, breast density, BMI, alcohol consumption, and smoking. Family history was defined as a prior diagnosis of breast cancer in a first-degree relative. For binary exposures (breastfeeding, OC use, MHT use, family history, alcohol, smoking), the comparison groups consisted of participants who did not have the exposure. For ordinal or continuous exposures (BMI, age at menarche, age at first live birth, duration of breastfeeding, duration of OC use, breast density), we considered the exposed category to be the one with highest degree of exposure, and the comparator category to be the one with lowest degree of exposure. For example, in the case of the continuous exposure, duration of breastfeeding, the exposed group had participants with highest duration of breastfeeding (≥12 months), and the comparison group had participants with least duration of breastfeeding (0 months).

### Data extraction and management

Five reviewers (NK, SE, SB, CF, and SAN) independently performed data extraction using a tabular template (Supplementary Figure 2, available online). This template captured information on authors, year of publication, study setting, study design, exposures, and outcome. For all ordinal and continuous exposures, we reconciled the level of exposure for exposed and comparison group for all studies at the extraction stage to enable pooling and meta-analysis. Whenever data were not available for exposures of interest in the desired format in the published material, we reached out to the corresponding authors to share the requisite data.

### Risk of bias

SE and CF independently assessed the study quality using the Newcastle Ottawa Quality Assessment Scale for case control studies and for cohort studies (17). Assessment entailed rating the studies on the basis of the following 3 elements for case control studies: participant selection procedures (case definition, representativeness of cases, cohort selection, and definition), comparability of cases and controls, and ascertainment of exposure (method of measurement, similarity of measurement method for cases and controls, nonresponse rate). For cohort studies, in addition to the previously described criteria for selection and comparability, the third component was ascertainment of outcome. This was assessed on the basis of the measurement method, length of follow-up, and lost to follow-up. Maximum possible score for selection component was 4, and comparability component could receive maximum score of 2. The third component of ascertainment of exposure/outcome could receive a maximum score of 3. The scores of all 3 components were converted into percentages for interpretation. An example of assessment of risk of bias is provided in Supplementary Figure 3 (available online).

### Statistical analysis

The association of exposures of interest with TNBC was quantified with weighted odds ratios (ORs). Weighting was done using a restricted maximum likelihood random effects model, as described by Hardy and Thompson (18). Heterogeneity was assessed by computing Higgins’s I^2^ statistic using Higgins and Thompson’s (19) method. For Higgins’s I^2^, we considered the values of I^2^ between 0% and 40% as low heterogeneity, 41% to 65% as moderate, and above 65% as high levels of statistical heterogeneity, provided the *P* value was less than .05. Sensitivity analysis was performed by carrying out iterations of the meta-analysis leaving one study out at a time, using the leave one out meta-analysis method. Continuity correction by adding a value of .5 to all the cells was performed at the estimation stage in studies that had any cells with a zero. Publication bias was assessed visually by contour enhanced funnel plots of log odds-ratio estimates and standard errors of individual studies. Quantitatively, small study effects were studied using Egger’s test. Statistical significance was assessed using 95% confidence intervals (CIs) for odds ratios, and *P* values for I^2^. Results were considered significant at *P* values less than .05. Only 2-sided *P* values have been used and reported in this article. All analyses were performed using STATA 18 (College Station, TX).

## Results

Our initial search yielded 1238 studies; after removal of 136 duplicates, 1102 studies were screened on the basis of title and abstract. A total of 89 studies from this list matched the inclusion criteria and underwent full-text screening. At this stage, from the reference lists of these studies and from a Google Scholar search, we identified 4 additional studies that matched the inclusion criteria and had the required estimates for meta-analysis. Thirty-three studies met inclusion criteria and were included in the meta-analysis (Table 1). The PRISMA flow diagram depicting inclusions and exclusions is present in Supplementary Figure 31 (available online).

**Table 1.** Characteristics of included studies^a^

ID	Authors	Year	Design	Location	Menopausal status	Sample size	# TNBC cases	Race specification	Exposures studied
1	Akinyemiju et al. (20)	2021	Case-control	Nigeria	Any	338	52	Black	BMI, MHT use
2	Ambrosone et al. (21)	2015	Case-control	USA	Any	36 304	1356	Black	Age at menarche, age at first live birth
3	Ambrosone et al. (22)	2014	Case-control	USA	Any	1146	131	Black	Breastfeeding, parity
4	Atkinson et al. (23)	2016	Case-control	USA	Any	620	64	Not specified	Breastfeeding, family history, parity
5	Azubuike et al. (24)	2022	Case-control	Nigeria	Any	759	71	Black	OC use, parity
6	Beaber et al. (25)	2014	Case-control	USA	Any	1867	171	Not specified	Duration of OC use, OC use
7	Bethea et al. (26)	2016	Case-control	USA	Any	21 940	696	Black	Family history
8	Bethea et al. (27)	2015	Case-control	USA	Any	12 935	494	Black	Duration of OC use, OC use
9	Bigman et al. (28)	2022	Case-control	Nigeria	Any	1400	123	Black	Alcohol, BMI, breastfeeding, parity, smoking
10	Ellingjord-Dale et al. (29)	2017	Case-control	Norway	Any	26 236	386	Not specified	Age at first live birth, breastfeeding, duration of breastfeeding, MHT use, OC use, parity
11	Figueroa et al. (30)	2020	Case-control	Ghana	Any	2198	102	Black	Parity
12	Gaudet et al. (31)	2011	Case-control	USA	Post-menopausal	3678	246	Not specified	BMI, family history, OC use
13	Gomes et al. (32)	2022	Case-control	Brazil	Any	634	43	Hispanic	Alcohol, BMI, family history, OC use, parity, smoking
14	Islam et al. (33)	2012	Case-control	Japan	Any	1415	68	East Asian	Age at first live birth, alcohol, BMI, breastfeeding, family history, parity
15	John et al. (34)	2018	Case-control	USA	Any	5669	558	Black/East Asian/Hispanic	Age at menarche, breastfeeding, duration of breastfeeding, parity
16	Kleinstern et al. (35)	2021	Case-control	USA	Any	13 640	369	Not specified	BMI, breast density, family history, MHT use
17	Klintman et al. (36)	2022	Cohort	Sweden	Post-menopausal	31 510	60	Not specified	BMI
18	Lee et al. (37)	2020	Case-control	Hong Kong	Any	1150	137	East Asian	BMI, duration of breastfeeding, parity
19	Li et al. (38)	2013	Case-control	USA	Any	1125	184	Not specified	Age at menarche, age at first live birth, breastfeeding, duration of breastfeeding, parity
20	Ma et al. (39)	2009	Case-control	USA	Post-menopausal	482	106	Not specified	Breast density
21	Ma et al. (40)	2017	Case-control	USA	Any	3002	554	Not specified	Age at menarche, age at first live birth, duration of breastfeeding, parity
22	Ma et al. (41)	2018	Case-control	USA	Post-menopausal	3416	1030	Not specified	BMI
23	McCarthy et al. (3)	2021	Cohort	USA	Any	198 278	300	Not specified	Age at menarche, age at first live birth, BMI, breast density, family history, parity
24	Palmer et al. (42)	2014	Case-control	USA	Not mentioned	14 747	567	Black	Breastfeeding, parity
25	Park et al. (43)	2016	Case-control	USA	Any	18 070	694	Black	Smoking
26	Phipps, Buist, et al. (44)	2011	Cohort	USA	Not mentioned	1 055 171	705	Not specified	Family history
27	Phipps et al. (45)	2011	Cohort	USA		155 723	307	Not specified	Age at menarche, age at first live birth, BMI, duration of breastfeeding, duration of OC use, family history, MHT use, OC use, parity, smoking
28	Phipps et al. (46)	2008	Case-control	USA	Post-menopausal	1554	78	Not specified	Age at menarche, age at first live birth, breastfeeding, family history, MHT use, parity
29	Razzaghi et al. (47)	2013	Case-control	USA	Any	1019	48	Not specified	Breast feeding, family history, OC use
30	Shin et al. (48)	2018	Case-control	Korea	Any	1883	31	East Asian	Breast density
31	Wang et al. (49)	2022	Cohort	USA	Post-menopausal	121 744	529	Not specified	Age at menarche, breastfeeding, BMI, family history, parity, smoking
32	Wang et al. (50)	2020	Case-control	China	Any	7974	448	East Asian	Age at menarche, age at first live birth, breastfeeding, duration of breastfeeding, parity
33	Williams et al. (51)	2016	Case-control	USA	Any	1495	229	Black	Alcohol

a BMI = body mass index; MHT = menopausal hormone therapy; OC = oral contraceptive; TNBC = triple-negative breast cancer.

### Overview of included studies

Of the 33 included studies (Table 1), 22 were from the United States, 4 from Africa (3 Nigeria, 1 Ghana), 4 from Asia (Japan, Hong Kong, China, and South Korea), 2 from Scandinavia (Norway and Sweden), and 1 from South America (Brazil). With respect to racial/ethnic distribution, 11 studies included exclusively Black participants, 4 Asian participants, 1 Hispanic participants, and the remaining 17 studies had participants from multiple racial/ethnic backgrounds. There were 4 cohort studies and 29 case-control studies included.

### Risk of bias

The summary of risk of bias assessment scores for all studies has been summarized in Supplementary Figure 4 (available online). Risk of bias assessment was performed for 3 elements in each study: 1) participant selection, 2) comparability of cases/controls, and 3) ascertainment of exposure/outcome. Most studies scored quite well in the comparability of cases and controls section, with a median score of 100%. The section on participant selection had a median score of 75% (interquartile range = 25%, 100%) indicating most studies had moderately good scores and were mostly free of selection bias. The section on exposure/outcome ascertainment had least scores compared to the other 2 sections with a median score of 34% (interquartile range = 34%, 67%).

### Age at first live birth

**Figure 1.**
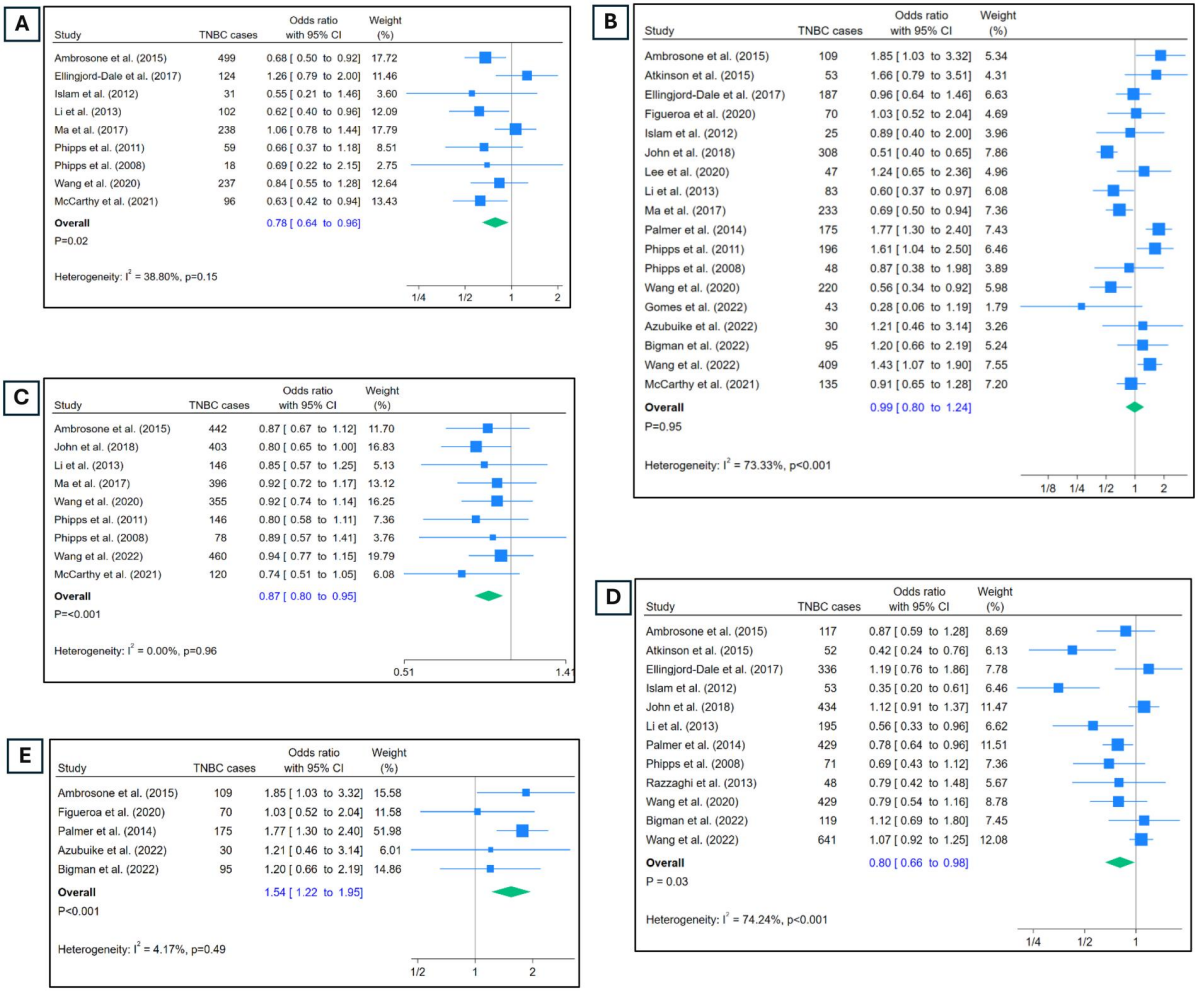
Odds of triple-negative breast cancer (TNBC) by reproductive risk factors. **A**) Odds of TNBC in those who had first live birth at 30 years of age or older vs those who had it at 19 years or younger. **B**) Odds of TNBC in those with 3 or more live births vs 0 live births. **C**) Odds of TNBC in those who had menarche at 14 years or older vs those who had it at 11 years or younger. **D**) Odds of TNBC in those who ever breastfed vs those who never did. **E**) Odds of TNBC in Black participants with 3 or more live births vs 0 live births.

Nine studies report data on the age at first live birth (Figure 1, A). Having a first birth at 30 years of age or older was associated with lower odds of TNBC compared with age 19 years or younger at first birth (OR = 0.78, 95% CI = 0.64 to 0.96). Heterogeneity in the estimates was low (I^2^ = 38%), and sensitivity analysis using leave-one-out meta-analysis method revealed there were no outliers in the included studies (Supplementary Figure 10, available online).

### Parity

Eighteen studies contributed estimates for parity (Figure 1, B). Participants with 3 or more live births had similar odds of developing TNBC compared with those had no live births (OR = 0.99, 95% CI = 0.8 to 1.24). There was high level of heterogeneity in the estimates (I^2^ = 73.3%, *P* < .00). From the sensitivity analysis, it appeared that considerable heterogeneity was contributed by 2 studies: John et al. (34) and Palmer et al. (42) (Supplementary Figure 5, A, available online). Omitting these 2 studies and running the meta-analysis again (Supplementary Figure 5, B, available online) reduced the heterogeneity to 57% while not changing the estimates much (OR = 1.01, 95% CI = 0.8 to 0.23). Restricting the analysis to studies with only Black participants (24,28,30,42) revealed highly significant association of 3 or more live births and occurrence of TNBC with OR = 1.54 and 95% CI: 1.22 to 1.95 (Figure 1, E). This also brought down the heterogeneity significantly to 4.17% (*P* = .49).

### Age at menarche

Nine studies reported odds of developing TNBC in those who experienced menarche at 14 years of age or older vs those who experienced it at 11 years of age or younger (Figure 1, C). Being older at menarche was associated with significantly lower odds of developing TNBC compared with those who were younger (OR = 0.87, 95% CI = 0.80 to 0.95). There was no heterogeneity in the estimates (I^2^ = 0%), and none of the studies were outliers (Supplementary Figure 9, available online).

### Breastfeeding

Exposure to breastfeeding in a binary form (ever/never) was reported by 12 studies (Figure 1, D). The odds of developing TNBC were significantly lower in those who reported as having ever breastfed vs those who reported never breastfeeding (OR = 0.80, 95% CI = 0.66 to 0.98). There was high level of heterogeneity in the estimates as indicated by an I^2^ of 74.2% (*P* < .01). As per the sensitivity analysis (Supplementary Figure 6, A, available online), omitting any study would not change the estimates or heterogeneity much (Supplementary Figure 6, B, available online).

### Duration of breastfeeding

Duration of breastfeeding was measured by 7 studies. The odds of developing TNBC were similar in participants who breastfed more than 12 months and those who never breastfed (Supplementary Figure 32, available online) with a pooled odds ratio of 1.02 (95% CI = 0.59 to 1.76). The level of heterogeneity in the estimates was high with I^2^ = 92.8%. Estimates from Phipps et al. (45) seemed to contribute sizeable heterogeneity; running the meta-analysis after omitting this study (Supplementary Figure 7, A and B, available online) reduced the heterogeneity to 53% and did not change the pooled estimate much (OR = 0.83, 95% CI = 0.66 to 1.05).

### OC use

Six studies contributed estimates for oral contraceptive use (Supplementary Figure 33, available online). Those who ever used OCs had similar odds of TNBC compared with those who never used them (OR = 1.16, 95% CI = 0.92 to 1.46). The estimates had moderate heterogeneity (I^2^ = 62.7%, *P* = .04). The heterogeneity was contributed mostly by Gaudet et al. (31) as assessed in sensitivity analysis (Supplementary Figure 11, A, available online), where removal of this study significantly improved the precision (Supplementary Figure 11, B, available online) as well as reduced the heterogeneity to 0% while not changing the overall effect estimate (OR = 1.18, 95% CI = 1.04 to 1.35, *P* = .012).

### Duration of OC use

**Figure 2.**
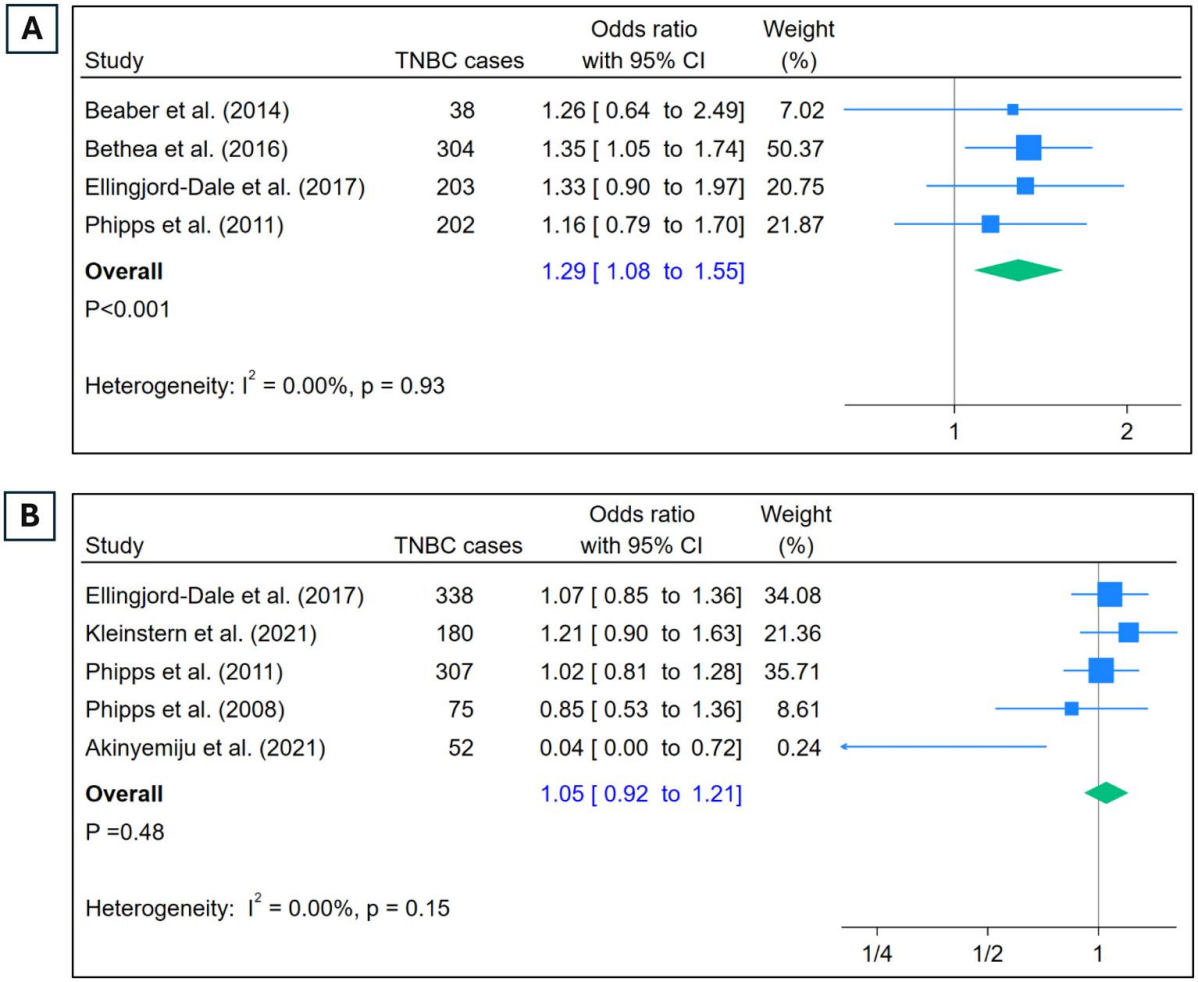
Odds of TNBC and use of hormones. **A**) Odds of TNBC in those with 10 or more years of OC use vs. no use. **B**) Odds of TNBC in those who ever used MHT vs those who never did. OC = oral contraceptive; MHT = menopausal hormone therapy; TNBC = triple-negative breast cancer.

Duration of OC use was reported by 4 studies (Figure 2, A). Comparison of odds of developing TNBC in those with 10 years or more of use of OCs vs those who never used OCs revealed a clear pattern of significantly increased odds in the users (OR = 1.29, 95% CI = 1.08 to 1.55). Sensitivity analysis did not indicate presence of outliers (Supplementary Figure 12, available online).

### Menopausal hormone therapy use

Five studies reported the use of MHT (Figure 2, B). The association between MHT use and TNBC was not statistically significant (OR = 1.05, 95% CI = 0.92 to 1.21). There was no heterogeneity in the estimates (I^2^ = 0%), and the sensitivity analysis did not indicate presence of outliers (Supplementary Figure 13, available online).

### Alcohol use

**Figure 3.**
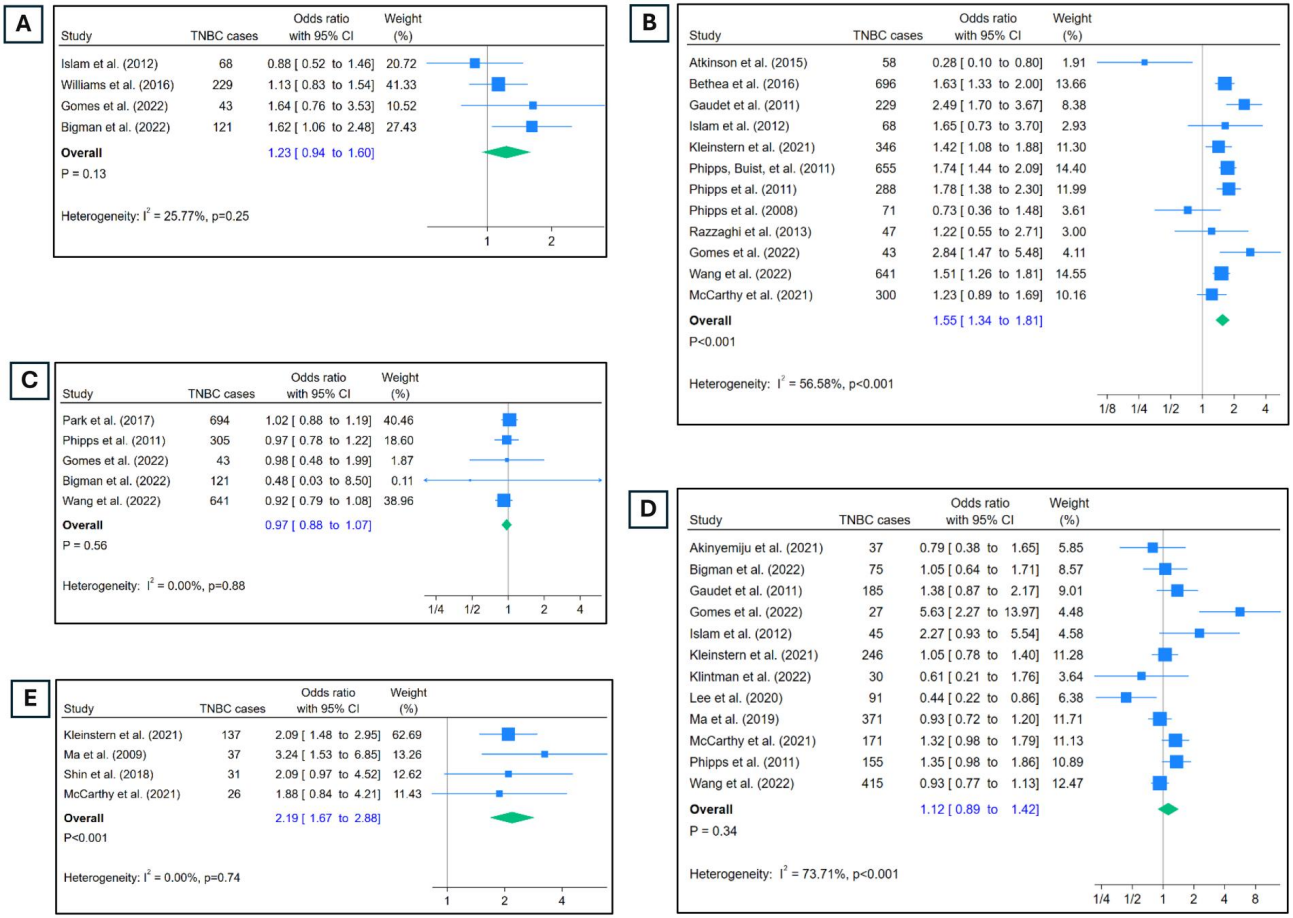
Odds of TNBC by lifestyle factors, family history, BMI, and breast density. **A**) Odds of TNBC in participants who ever consumed alcohol vs those who never did. **B**) Odds of TNBC in those with history of breast cancer in 1° relative vs those without. **C**) Odds of TNBC in ever smokers vs never smokers. **D**) Odds of TNBC in those with BMI ≥30 kg/m^2^ vs BMI <25 kg/m^2^, stratified by menopausal status. **E**) Odds of TNBC in those with ≥75% mammographic density vs ≤25%. BMI = body mass index.

Use of alcohol was analyzed in a binary form as “ever used” vs “never used” (Figure 3, A). This comparison did not reveal any difference in the odds of developing TNBC (OR = 1.23, 95% CI = 0.94 to 1.6). Heterogeneity in the estimates was low (I^2^ = 25%), and there were no significant outliers (Supplementary Figure 14, available online).

### Family history

Twelve studies contributed estimates on family history of breast cancer (Figure 3, B). Participants with family history of breast cancer in an immediate family member had significantly higher odds of developing TNBC compared with those who did not (OR = 1.55, 95% CI = 1.34 to 1.81). There was moderate heterogeneity in the estimates (I^2^ = 56.5%, *P* < .01). Although 2 studies reported a beneficial effect of having family history on developing TNBC [Atkinson et al. (23) and Phipps et al. (46)], sensitivity analysis did not indicate these to significantly influence the overall estimate (Supplementary Figure 8, available online).

### Smoking

Among 5 studies, participants who reported ever smoking had similar odds of developing TNBC (Figure 3, C) compared with those who reported being never smokers (OR = 0.97, 95% CI = 0.88 to 1.07). Estimates did not have heterogeneity (I^2^ = 0%), and sensitivity analysis did not reveal presence of significant outliers (Supplementary Figure 15, available online).

### BMI

Overall, estimates on BMI and TNBC were contributed by 12 studies (Figure 3, D). The pooled estimates revealed that a BMI of 30 or higher at diagnosis was not associated with TNBC (OR = 1.12, 95% CI = 0.89 to 1.42). The level of heterogeneity was high (I^2^ = 73.7%, *P* < .01), and one particular study, Gomes et al. (32), contributed a significant portion of it. Upon omitting estimates from this study (Supplementary Figure 17, A and B, available online), the heterogeneity reduced significantly to 30%, and the pooled odds ratio did not change significantly (OR = 1.03, 95% CI = 0.88 to 1.21). Restricting the analysis to post-menopausal women only (3,31,36,41,45,49) did not change the estimate much (OR = 1.07, 95% CI = 0.87 to 1.30). Higher BMI was found not be associated with TNBC within postmenopausal women or group of mixed menopausal status women.

### Mammographic breast density

Among 4 studies, higher breast density was significantly associated with higher odds of TNBC (Figure 3, E; OR = 2.19, 95% CI = 1.67 to 2.88). There was no heterogeneity (I^2^ = 0%) or presence of outliers (Supplementary Figure 16, available online).

### Publication bias

Visual asymmetry in contour-enhanced funnel plots was not seen in any of the exposures, except in OC use (Supplementary Figures 18-30, available online). However, Egger’s test for presence of small-study effects was not significant in any of the exposures, except breastfeeding (Supplementary Figure 19, A, available online) and MHT use (Supplementary Figure 25, A, available online) with *P* = .016 and *P* = .025, respectively. Assessing the extent of missing studies in both these using the trim and fill method indicated 2 studies being imputed for breastfeeding with observed 12 studies (Supplementary Figure 19, B, available online). For MHT as well, the trim and fill algorithm indicated imputation of 2 missing studies with observed 5 studies (Supplementary Figure 25, B, available online).

## Discussion

This systematic review and meta-analysis examined the association of established breast cancer risk factors with TNBC. Family history, longer duration of oral contraceptive use, and higher breast density were significantly associated with increased risk for TNBC, whereas later age at menarche and breastfeeding were protective against TNBC (Table 2). In contrast to published associations with overall breast cancer, later age at first birth was protective against TNBC. Parity, MHT, alcohol, smoking, and BMI were not significantly associated with risk for TNBC. These findings further clarify differences in etiology between TNBC and breast cancer overall and highlight the need to identify additional actionable risk factors for TNBC to improve prevention and early detection of these poor prognosis breast tumors.

**Table 2.** Summary of findings^a^

Exposure	Pooled OR (95% CI) for TNBC	Heterogeneity I^2^ (P value)	# Studies	#TNBC cases
*Reproductive Risk Factors*				
Parity (≥3 live births/0 live births)	0.99 (0.80, 1.24)	73% (<.001)	18	2466
Breastfeeding (Yes/No)	0.80 (0.66, 0.98)	74% (<.001)	8	2924
Duration of breastfeeding (≥12 m/0 m)	1.02 (0.59, 1.76)	92% (<.001)	7	1294
Age at menarche (≥14 yr/<12 yr)	0.87 (0.80, 0.95)	0% (.96)	9	2546
Age at first live birth (≥30 yr/<20 yr)	0.78 (0.64, 0.96)	38% (.15)	9	1404
*Hormone use*				
OC use (Yes/No)	1.16 (0.92, 1.46)	62% (.04)	6	1310
Duration of OC use (≥10 yr/0 yr)	1.29 (1.08, 1.55)	0% (.93)	4	747
MHT use (Yes/No)	1.05 (0.92, 1.21)	0% (.15)	5	952
*Other Factors*				
Family history (Yes/No)	1.55 (1.34, 1.81)	56% (<.001)	12	3442
Alcohol (Yes/No)	1.23 (0.94, 1.60)	25% (.25)	4	461
Smoking (Yes/No)	0.97 (0.88, 1.07)	0% (.88)	5	1804
BMI (≥30/<25)	1.12 (0.89, 1.42)	73% (<.001)	12	1848
Breast density (≥75%/≤25%)	2.19 (1.67, 2.88)	0% (0.74)	4	231

a CI = confidence intervals; TNBC = triple negative breast cancer; BMI = body mass index; OC = oral contraceptive; MHT = menopausal hormone therapy.

Both family history and breast density were strongly associated with increased risk of TNBC, with similar associations to those reported for breast cancer overall (9-11). This suggests that breast cancer prevention and screening recommendations targeted to patients with family history and dense breasts are relevant for TNBC prevention and early detection. Similarly, later age at menarche and ever breastfeeding were associated with reduced risk of TNBC, similar to breast cancer overall (12). We found no significant association between parity and TNBC overall, but having high parity was associated with increased TNBC risk among Black women. This finding is consistent with prior findings from the Black Women’s Health Study that that TNBC risk was particularly high among women with high parity who had not breastfed (42). However, we were unable to evaluate the interaction between parity and breastfeeding, which has been previously reported (34,42), as these data were unavailable in most studies (42).

We found use of OCs for at least 10 years was associated with nearly 30% increased risk of TNBC. Studies have consistently shown a small but significant increased risk of breast cancer overall due to OC use, which is highest when patients are using OCs and declines with increased time since last use of OCs (52,53). The level of risk estimated in this study for long-term OC use is higher than what was reported for the risk of long-term OC use with breast cancer overall in a meta-analysis of cohort studies (54), but consistent with a large study of OC use and breast cancer risk overall among 1.8 million Danish women (55).

Contrary to studies in breast cancer overall, having a first child at age 30 or older decreased risk for TNBC by more than 20% compared with early age at first birth (<20). Maternal ages at first birth have been increasing, with most recent data showing a median age at first birth in the United States of 30 years overall, and only slightly lower, age 28, for Black women (13). These reproductive patterns would suggest decreasing risk for TNBC over time on the population level.

We observed no statistically significant association between BMI and risk of TNBC, either overall or when limiting to studies of postmenopausal women. This is in direct contrast to the well-established positive association between higher BMI and risk of postmenopausal breast cancer overall and ER/PR+ breast cancers (14). A prior meta-analysis similarly found no association between postmenopausal BMI and hormone receptor negative tumors (14). There were too few studies with enough information to evaluate the association of BMI and TNBC among premenopausal women.

Black women in the United States have significantly higher rates of TNBC than White women (56). Black women on average have earlier age at menarche (57), are an earlier age at first birth (13), and are less likely to breastfeed (58) than White women. All of these risk factor distributions are consistent with observed higher population rates of TNBC for Black women compared with White women. Although age at menarche is not easily modifiable, recent trends toward older age at first birth as well as interventions to increase breastfeeding rates among Black women may help reduce risk of TNBC.

### Strengths and limitations

This is the first comprehensive meta-analysis to report the association of 13 known risk factors of breast cancer with TNBC and, to our knowledge, the largest meta-analysis of risk factors for TNBC published to date. One of the main strengths of this analysis is the large sample size in all the included analyses and, consequently, the precision in the estimates.

Of the 13 exposures meta-analyzed in this article, most had low to moderate levels of heterogeneity. Even among the exposures with higher levels of heterogeneity, the heterogeneity went down to 30% for BMI and 53% for duration of breastfeeding upon excluding 1 study. For breastfeeding, however, the heterogeneity was more spread out across the studies. This is possibly because of the broad definition of breastfeeding categories as “ever” and “never.” Owing to this binary categorization, most participants who fell under the “ever” category would still likely have varying levels of breastfeeding—this is bound to introduce between-study differences in the estimates.

One limitation of the existing literature was the dearth of studies that stratified effect estimates by menopause status or age at menopause, making evaluations of differences in risk factor associations by menopause status, particularly among premenopausal women, difficult. Given that only 2 (25,31) of the included studies reported the formulation of OCs used, it was not possible to examine the effects of different formulations of OCs. Similarly, we were unable to evaluate the effects of duration and formulation of MHT use. In addition, there was a lack of sufficient data to evaluate risk factor associations by race/ethnicity. Of the included studies, only 2 (3,40) reported race stratified estimates for age at first live birth, breastfeeding, and parity, which was not sufficient to meta-analyze. The presence of *BRCA1* mutation is associated with increased risk of TNBC. With the exception of McCarthy et al. (3), who excluded *BRCA1* carriers, none of the included studies disclosed the *BRCA1* status of the participants. Consequently, it is not possible to completely rule out the potential effect of *BRCA1* mutations in the estimates. All included studies except 2 (35 and 41) excluded participants with secondary breast cancer. Because these two studies contributed estimates for parity and OC use, it cannot be ruled out that associations seen are partly because of inclusion of secondary breast cancer cases. Finally, given the pooled estimates were computed using unadjusted count data extracted from individual studies, it was not possible to compute adjusted pooled estimates. Because of this, the strength of associations may be overstated.

In conclusion, our findings confirm family history, breast density, and longer use of oral contraceptive hormones are associated with increased odds of TNBC. Age at first birth was inversely associated with TNBC, which is contrary to the association seen with breast cancer overall. We did not find a significant association of parity, MHT, alcohol, smoking, and BMI with overall TNBC risk; however, a significant association of high parity with TNBC among Black women was seen. Given the new insight from this study that TNBC has a unique risk-factor profile compared with breast cancer overall, this should inform screening and risk reduction strategies. Further research should evaluate whether additional lifestyle and environmental risk factors such as diet, physical activity, environmental exposures, and medication use contribute to TNBC risk in order to design policy interventions for prevention.

## Supplementary Material

djae056_Supplementary_Data

## Data Availability

No new/primary data were generated for this work. The data extracted for conducting the analysis presented in this work will be available upon reasonable request.
