# Supplementary material for: The unique risk factor profile of triple-negative breast cancer: a comprehensive meta-analysis
Source: J Natl Cancer Inst. 2024 Mar 5;116(8):1210–9. doi: 10.1093/jnci/djae056 (PMC11308168; doi:10.1093/jnci/djae056)

SUPPLEMENTARY MATERIAL

Supplementary Figure 1: Detailed Search Queries

Database	Search Query
Medline	(((("Breast Neoplasms"[Mesh]) OR "Breast Cancer") AND ("Triple Negative Breast Neoplasms"[Mesh] OR "luminal" OR "basal-like")) AND (((((("Menarche"[Mesh]) OR "Menopause"[Mesh]OR "menopausal" OR "age at first birth") OR "Parity"[Mesh]) OR "Breast Feeding"[Mesh]) OR "Hormone Replacement Therapy"[Mesh]) OR "Estrogen Replacement Therapy"[Mesh]OR "HRT" OR "postmenopausal hormone" OR "hormone therapy" OR "estrogen therapy")) AND ((((((("Contraceptives, Oral"[Mesh]) OR "Medical History Taking"[Mesh]) OR "Fibrocystic Breast Disease"[Mesh]) OR "Body Mass Index"[Mesh]) OR "Obesity"[Mesh]) OR "Alcohols"[Mesh]) OR "Breast Density"[Mesh] OR "mammographic density")
EMBASE	'breast cancer'/exp OR 'breast tumor'/exp OR 'triple negative breast cancer'/exp AND 'menarche'/exp OR 'age at menarche'/exp OR 'parity'/exp OR 'obesity'/exp OR 'alcohol consumption'/exp OR 'oral contraceptive use'/exp OR 'breast feeding duration'/exp OR 'menopause'/exp OR 'breast density'/exp OR 'hormone substitution'/exp OR 'family history'/exp OR 'biopsy'/exp AND [female]/lim AND [adult]/lim AND ('Article'/it OR 'Article in Press'/it) AND ('case control study'/de OR 'cohort analysis'/de OR 'controlled clinical trial'/de OR 'controlled study'/de OR 'longitudinal study'/de OR 'population based case control study'/de OR 'prospective study'/de OR 'randomized controlled trial'/de OR 'retrospective study'/de)
SCOPUS	(TITLE-ABS-KEY (breast AND cancer OR breast AND neoplasm OR triple AND negative AND breast AND cancer) AND TITLE-ABS-KEY ("Menarche" OR "Parity" OR "Adiposity" OR "Alcohol Drinking" OR "Contraceptives, Oral" OR "Breast Feeding" OR "Menopause" OR "Breast Density" OR "Hormone Replacement Therapy" OR age AND at AND menarche OR age AND at AND menopause OR biopsy AND history))

Supplementary Figure 2: Data Extraction Template

	A	B	C	D	E	F	G	H	I	J	K	L	M
1	Study ID	Exposure	Unit	Authors, Title	Design	Duration (days)	Location	Exposure level in "Exposed"	Exposure level in "Non-exposed"	"Exposed" group N	"Non-Exposed" group N	Exposed # of outcome events	"Non-exposed" # of outcome events
2	Study 1	Exposure A											
3	Study 1	Exposure B											
4	Study 1	Exposure C											
5	Study 2	Exposure B											
6	Study 2	Exposure C											
7													
8													
9													
10													
11													
12													
13													
14													
15													
16													
17													
18													

Supplementary Figure 3: Example of risk of bias Assessment for the study Akinyemiju et al 2021

Risk of Bias Assessment

Scale/Tool: Newcastle - Ottawa Quality Assessment Scale for Case Control Studies

Study Authors: Akinyemiju et al 2021

Study Title: Association of body composition with odds of breast cancer by molecular subtype: analysis of the mechanisms for established and novel risk factors for breast cancer in Nigerian women (MEND) study

Scoring criteria: A study can be awarded a maximum of one star (1 point) for each numbered item within the Selection and Exposure categories. A maximum of two stars (2 points) can be given for Comparability.

Section A: Selection

1) Is the case definition adequate?
   a) yes, with independent validation (if cases come from registry, laboratory data)
   b) yes, eg record linkage or based on self-reports
   c) no description

2) Representativeness of the cases
   a) consecutive or obviously representative series of cases (star if cases come from geographic area or hospital)
   b) not stated
3) Selection of Controls
   a) community controls * (1 point)
   b) hospital controls
   c) no description
4) Definition of Controls
   a) no history of disease (endpoint) * (1 point)
   b) no description of source

Section B: Comparability

1) Comparability of cases and controls on the basis of the design or analysis
   a) study controls for Age * (1 point)
   b) study controls for additional factors (sociodemographic characteristics, reproductive history, and self and family history of cancer; anthropometric measurements) * (1 point)

Section C: Exposure

1) Ascertainment of exposure
   a) secure record (eg surgical records)
   b) structured interview where blind to case/control status
   c) interview not blinded to case/control status
   d) medical record only
   e) no description
2) Same method of ascertainment for cases and controls
   a) yes *(1 point)
   b) no
3) Non-Response rate
   a) same rate for both groups (10% or less difference)
   b) non respondents described
   c) rate different and no designation

SCORES: **Selection = 2 out of 4 (50%), Comparability = 2 out of 2 (100%), Exposure ascertainment = 1 out of 3 (0.33%)**

Supplementary Figure 4: Summary of Risk of Bias Assessment Scores for Included Studies

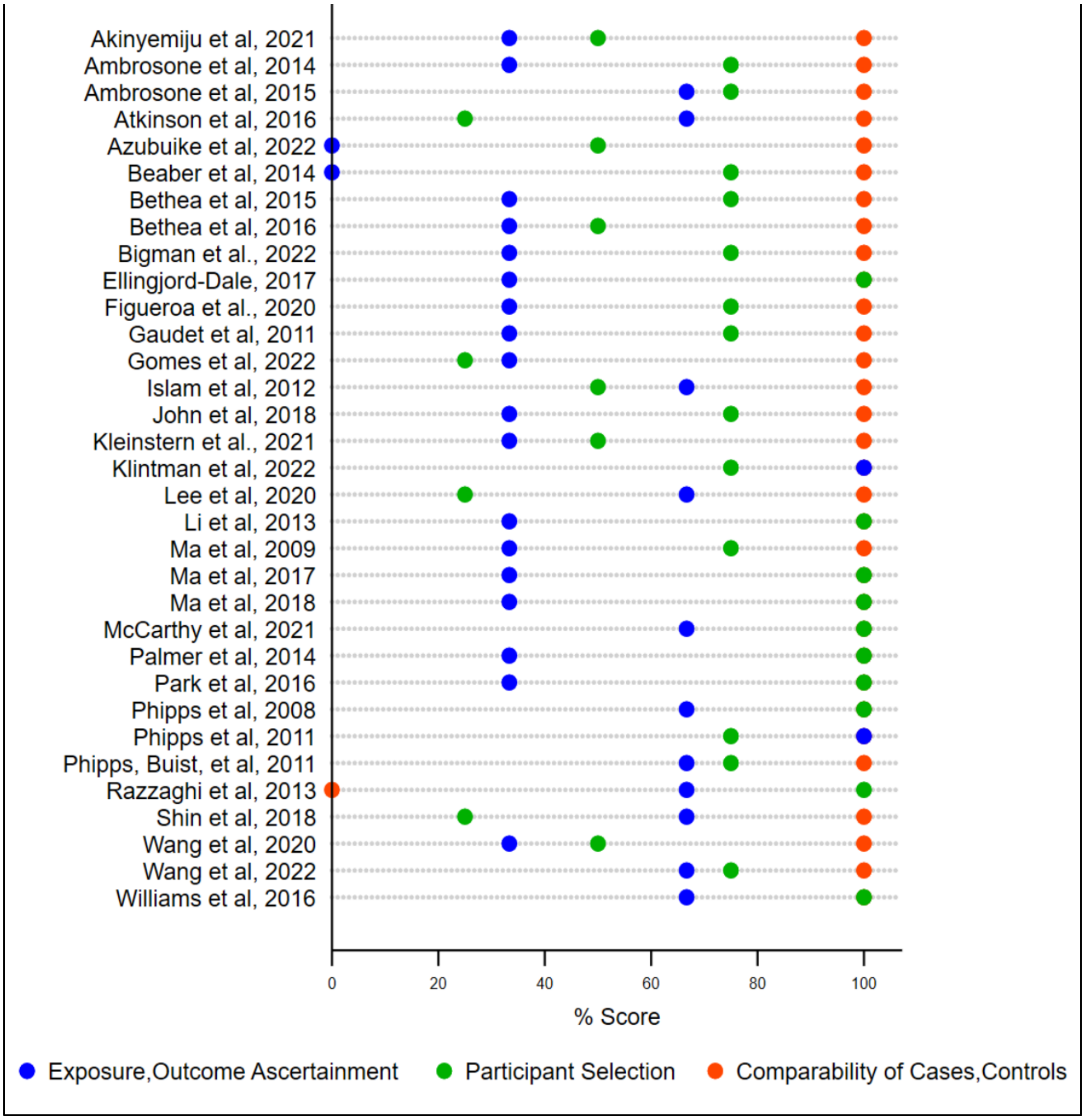

Supplementary Figure 5 - a: Leave One Out Sensitivity Analysis for Parity

Omitted study	n11	n12	n21	n22	Odds ratio with 95% CI	p-value
Ambrosone et al 2015	95	614	14	167	0.96 [0.77, 1.20]	0.713
Atkinson et al 2015	44	353	9	120	0.97 [0.78, 1.21]	0.795
Ellingjord-Dale et al 2017	153	673	34	144	1.00 [0.79, 1.26]	0.972
Figueroa et al 2020	60	1332	10	228	0.99 [0.79, 1.25]	0.945
Islam et al 2012	14	307	11	215	1.00 [0.80, 1.25]	0.987
John et al 2018	184	2164	124	746	1.05 [0.86, 1.30]	0.611
Lee et al 2020	32	290	15	169	0.98 [0.78, 1.23]	0.876
Li et al 2013	32	198	51	188	1.03 [0.82, 1.28]	0.814
Ma et al 2017	165	882	68	250	1.02 [0.81, 1.28]	0.845
Palmer et al 2014	102	2140	73	2709	0.95 [0.76, 1.18]	0.623
Phipps, Chlebowski, Prentice, et al 2011	173	81558	23	17509	0.96 [0.77, 1.20]	0.725
Phipps, et al 2008	41	877	7	130	1.00 [0.80, 1.25]	0.994
Wang et al 2020	200	1952	20	109	1.03 [0.83, 1.28]	0.789
Gomes et al 2022	2	48	41	273	1.02 [0.82, 1.26]	0.882
Azubuike et al 2022	24	146	6	44	0.99 [0.79, 1.24]	0.910
Bigman et al 2022	81	564	14	117	0.98 [0.78, 1.24]	0.885
Wang et al 2022	356	69451	53	14749	0.96 [0.77, 1.21]	0.757
McCarthy et al 2021	66	43049	69	40963	1.00 [0.79, 1.26]	0.998

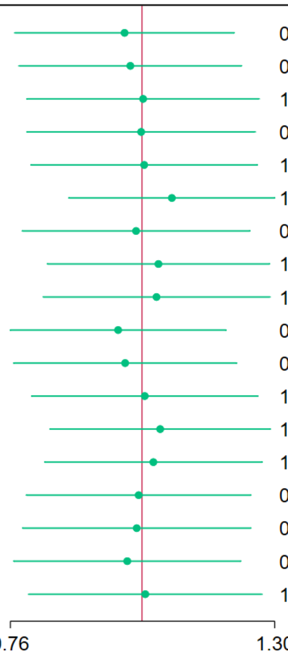

Supplementary Figure 5 - b: Forest plot for Parity omitting Palmer et al 2014, John e al 2018

Study	TNBC cases	Odds ratio with 95% CI	Weight (%)
Ambrosone et al 2015	109	1.85 [1.03, 3.32]	6.11
Atkinson et al 2015	53	1.66 [0.79, 3.51]	4.64
Ellingjord-Dale et al 2017	187	0.96 [0.64, 1.46]	8.22
Figueroa et al 2020	70	1.03 [0.52, 2.04]	5.16
Islam et al 2012	25	0.89 [0.40, 2.00]	4.19
Lee et al 2020	47	1.24 [0.65, 2.36]	5.55
Li et al 2013	83	0.60 [0.37, 0.97]	7.28
Ma et al 2017	233	0.69 [0.50, 0.94]	9.57
Phipps et al 2011	196	1.61 [1.04, 2.50]	7.92
Phipps, et al 2008	48	0.87 [0.38, 1.98]	4.09
Wang et al 2020	220	0.56 [0.34, 0.92]	7.11
Gomes et al 2022	43	0.28 [0.06, 1.19]	1.69
Azubuike et al 2022	30	1.21 [0.46, 3.14]	3.31
Bigman et al 2022	95	1.20 [0.66, 2.19]	5.95
Wang et al 2022	409	1.43 [1.07, 1.90]	9.94
McCarthy et al 2021	135	0.91 [0.65, 1.28]	9.26
**Overall**		1.01 [0.82, 1.23]	

p=0.96

Heterogeneity: I^2 = 57.03%, p<0.001

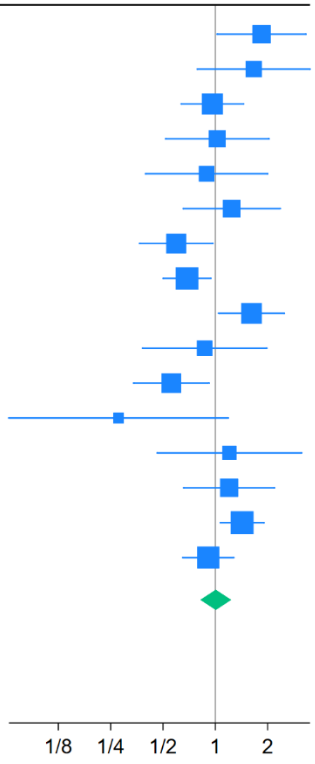

Supplementary Figure 6 - a: Leave One Out Sensitivity Analysis for Breastfeeding (ever vs. never)

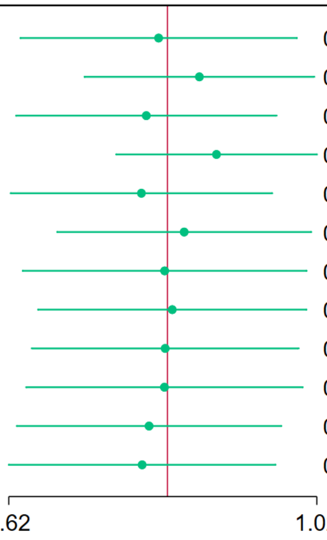

Omitted study	n11	n12	n21	n22	Odds ratio with 95% CI	p-value
Ambrosone et al 2015	52	406	65	442	0.79 [0.64, 0.99]	0.040
Atkinson et al 2015	23	279	29	149	0.85 [0.71, 1.02]	0.076
Ellingjord-Dale et al 2017	312	1479	24	135	0.78 [0.63, 0.96]	0.018
Islam et al 2012	26	875	27	319	0.87 [0.74, 1.02]	0.090
John et al 2018	272	2623	162	1742	0.77 [0.63, 0.95]	0.015
Li et al 2013	176	993	19	60	0.83 [0.68, 1.01]	0.066
Palmer et al 2014	160	3364	269	4422	0.80 [0.64, 1.01]	0.055
Phipps, et al 2008	40	875	31	471	0.81 [0.65, 1.00]	0.056
Razzaghi et al 2013	16	204	32	324	0.80 [0.65, 0.99]	0.042
Wang et al 2020	397	3829	32	244	0.80 [0.64, 1.00]	0.049
Bigman et al 2022	95	677	24	191	0.78 [0.63, 0.97]	0.022
Wang et al 2022	344	67481	297	62563	0.77 [0.62, 0.96]	0.018

Supplementary Figure 6 – b: Forest Plot for Breastfeeding after omitting Islam et al 2012 and Atkinson et al 2015

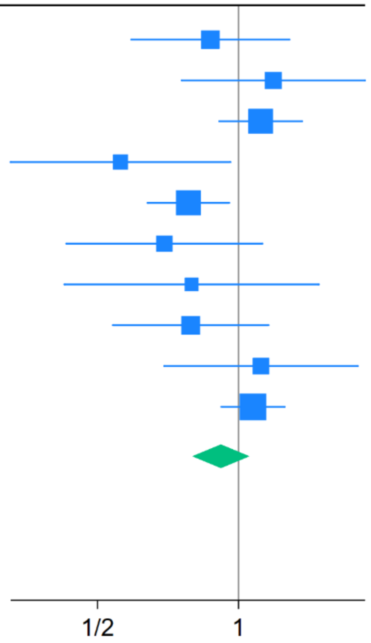

Study	TNBC cases	Odds ratio with 95% CI	Weight (%)
Ambrosone et al 2015	117	0.87 [0.59, 1.28]	8.65
Ellingjord-Dale et al 2017	336	1.19 [0.76, 1.86]	7.03
John et al 2018	434	1.12 [0.91, 1.37]	16.68
Li et al 2013	195	0.56 [0.33, 0.96]	5.34
Palmer et al 2014	429	0.78 [0.64, 0.96]	16.85
Phipps, et al 2008	71	0.69 [0.43, 1.12]	6.37
Razzaghi et al 2013	48	0.79 [0.42, 1.48]	4.21
Wang et al 2020	429	0.79 [0.54, 1.16]	8.83
Bigman et al 2022	119	1.12 [0.69, 1.80]	6.50
Wang et al 2022	641	1.07 [0.92, 1.25]	19.55
**Overall**		0.92 [0.80, 1.05]	

p=0.23

Heterogeneity: I^2 = 45.51%, p=0.06

Supplementary Figure 7-a: Leave One Out Sensitivity Analysis for Duration of Breastfeeding

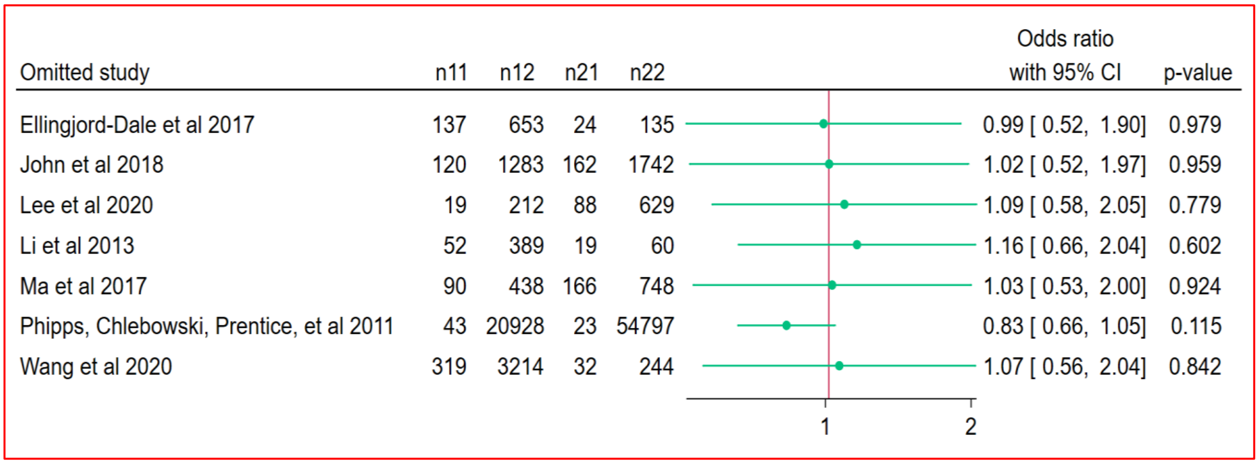

Supplementary Figure 7-b: Forest Plot for Duration of Breastfeeding after omitting Phipps et al 2011

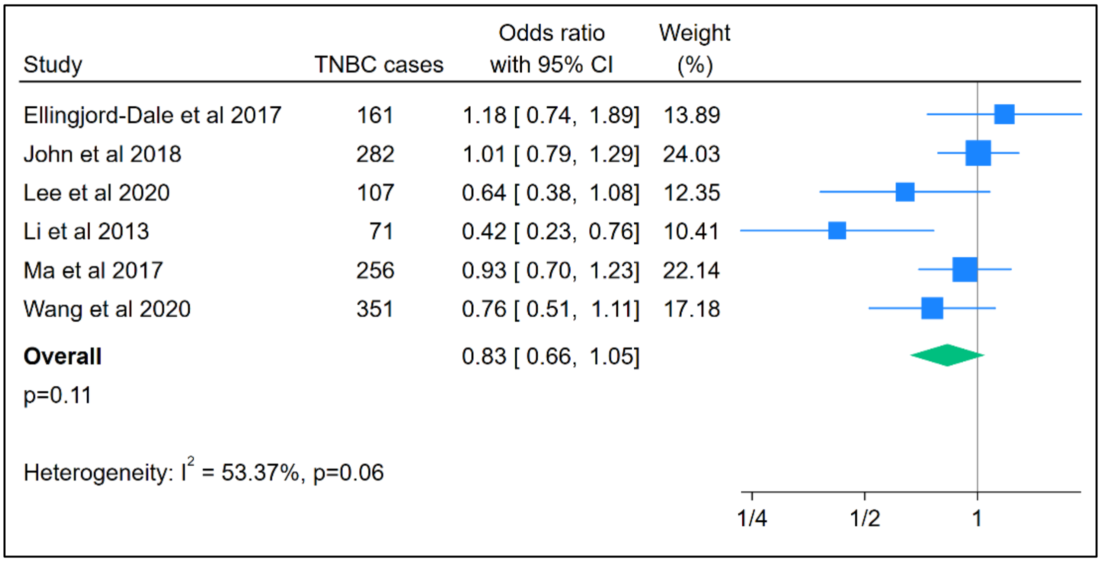

Supplementary Figure 8: Leave One Out Sensitivity Analysis for Family History

Omitted study	ed	enond	noned	nonenond	Odds ratio with 95% CI	p-value
Atkinson et al 2015	4	112	54	428	1.61 [1.45, 1.79]	0.000
Bethea et al 2016	112	2236	584	19008	1.50 [1.21, 1.87]	0.000
Gaudet et al 2011	35	218	194	3013	1.55 [1.40, 1.70]	0.000
Islam et al 2012	7	92	61	1320	1.54 [1.30, 1.83]	0.000
Kleinstern et al 2021	63	1366	283	8725	1.54 [1.25, 1.89]	0.000
Phipps, Buist, Malone, et al 2011	144	132598	511	816421	1.49 [1.21, 1.85]	0.000
Phipps, Chlebowski, Prentice, et al 2011	81	25648	207	116663	1.49 [1.22, 1.84]	0.000
Phipps, et al 2008	9	230	62	1151	1.61 [1.43, 1.80]	0.000
Razzaghi et al 2013	8	74	39	440	1.56 [1.33, 1.83]	0.000
Gomes et al 2022	19	70	24	251	1.52 [1.32, 1.76]	0.000
Wang et al 2022	157	22482	484	104862	1.52 [1.22, 1.89]	0.000
McCarthy et al 2021	45	24277	255	168841	1.62 [1.47, 1.80]	0.000

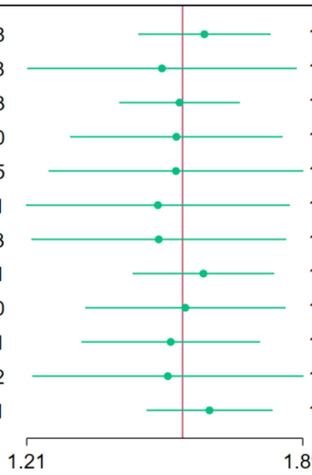

Supplementary Figure 9: Leave One Out Sensitivity Analysis for Age at Menarche

Omitted study	n11	n12	n21	n22	Odds ratio with 95% CI	p-value
Ambrosone et al 2015	71	2005	371	9063	0.87 [0.79, 0.96]	0.004
John et al 2018	139	1499	264	2290	0.88 [0.80, 0.97]	0.013
Li et al 2013	102	523	44	191	0.87 [0.80, 0.95]	0.003
Ma et al 2017	107	522	289	1292	0.86 [0.79, 0.95]	0.002
Wang et al 2020	183	1746	172	1505	0.86 [0.78, 0.95]	0.002
Phipps, Chlebowski, Prentice, et al 2011	67	34660	79	32816	0.88 [0.80, 0.96]	0.005
Phipps, et al 2008	41	817	37	658	0.87 [0.79, 0.95]	0.002
Wang et al 2022	141	28756	319	61156	0.85 [0.77, 0.94]	0.002
McCarthy et al 2021	63	46421	57	30899	0.88 [0.80, 0.96]	0.006

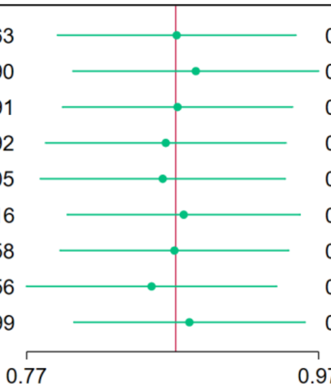

Supplementary Figure 10: Leave One Out Sensitivity Analysis for Age at 1st Live Birth

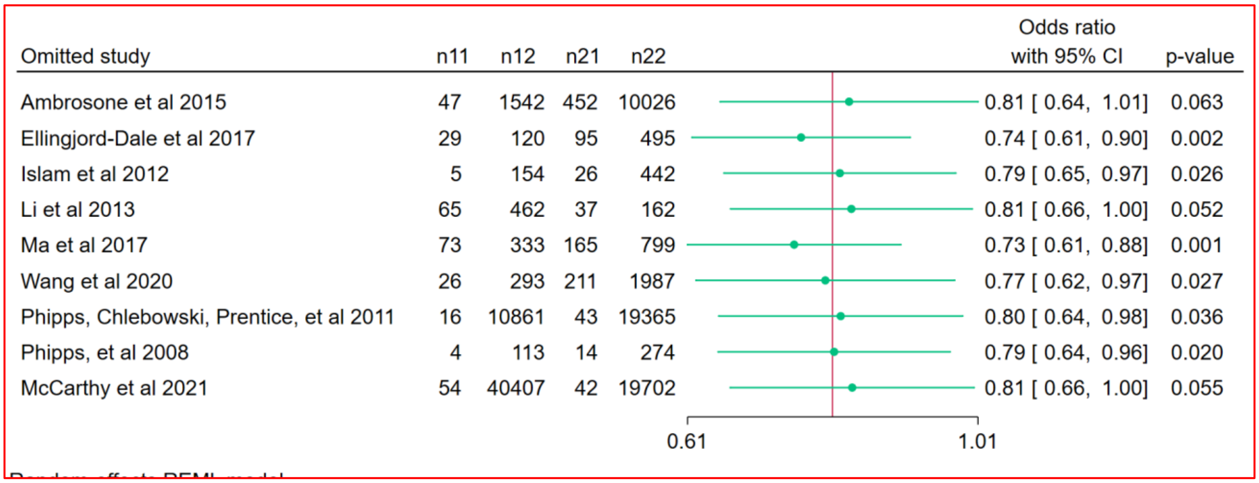

Supplementary Figure 11a: Leave One Out Sensitivity Analysis for Oral Contraceptive Use

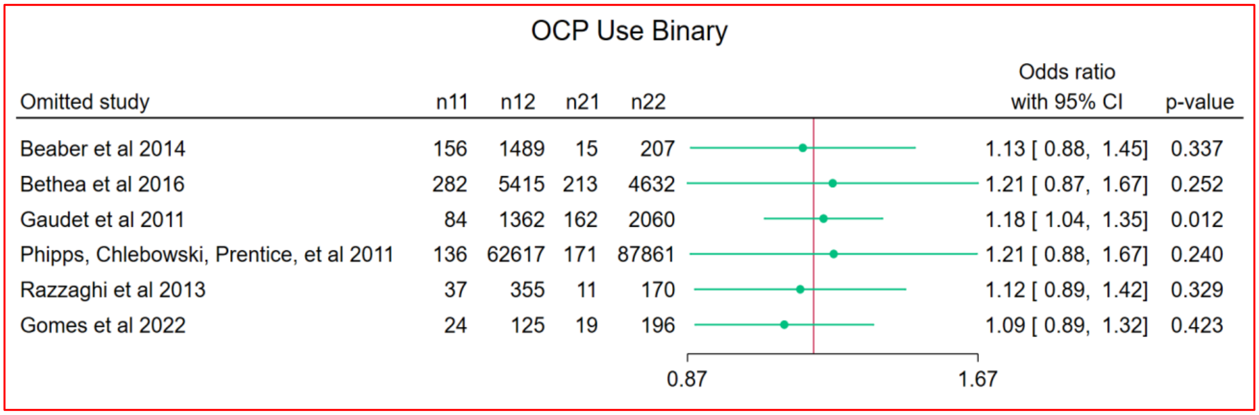

Supplementary Figure 11b: Forest Plot for OCP Use after omitting Phipps et al 2011

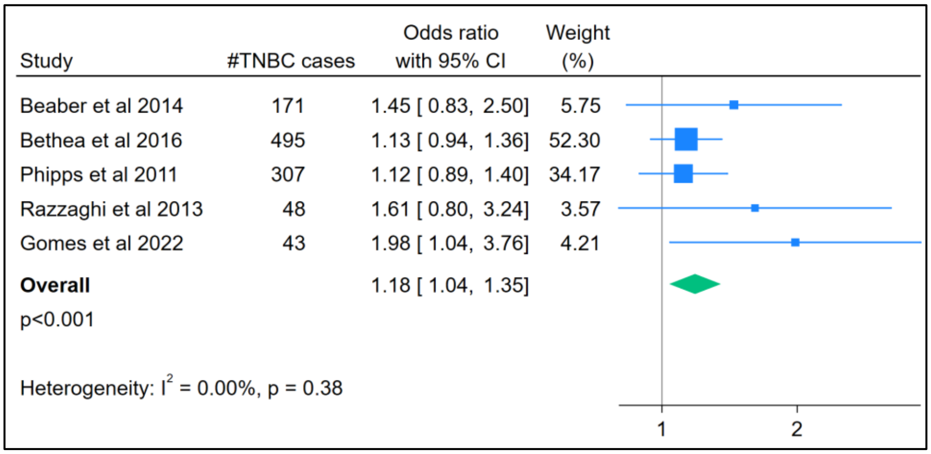

Supplementary Figure 12: Leave One Out Sensitivity Analysis for Duration of Oral Contraceptive Use

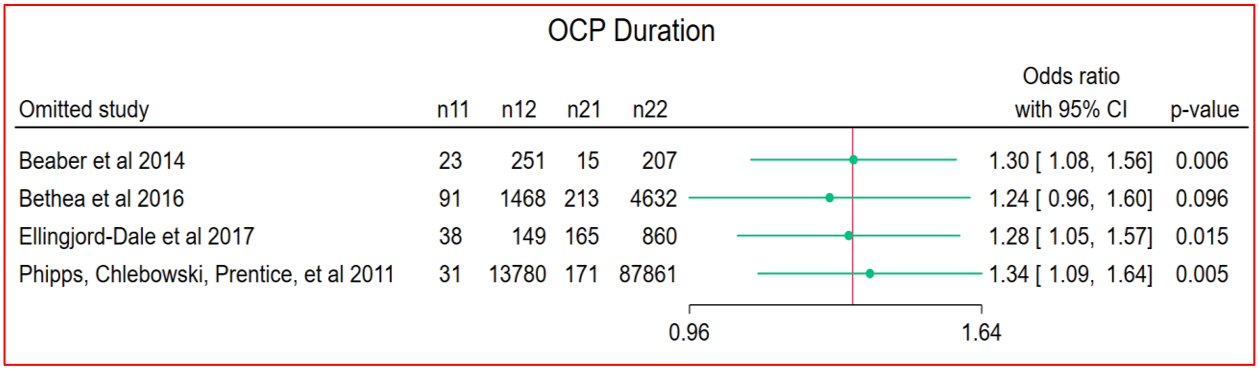

Supplementary Figure 13: Leave One Out Sensitivity Analysis for MHT Use

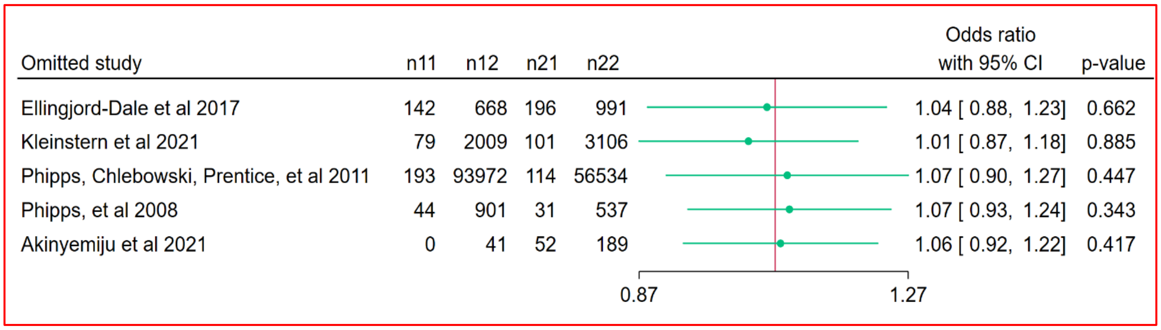

Supplementary Figure 14 Leave One Out Sensitivity Analysis for Alcohol Use

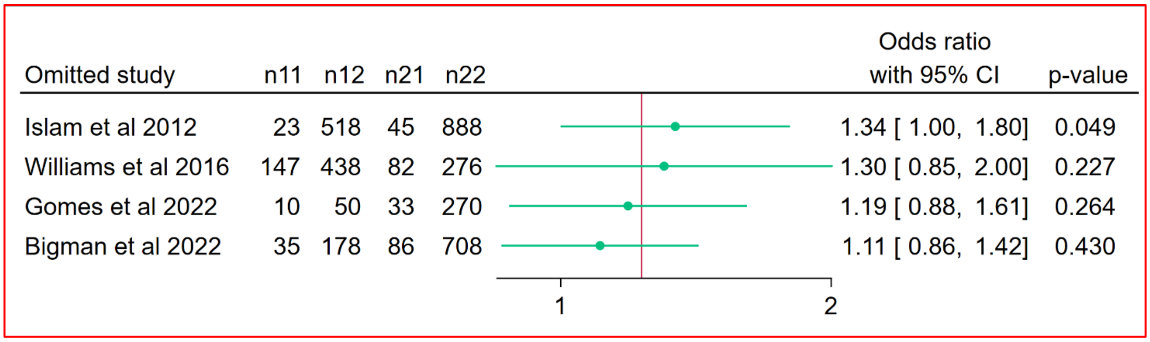

Supplementary Figure 15 Leave One Out Sensitivity Analysis for Smoking

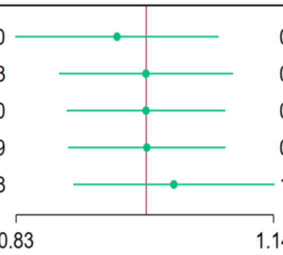

Omitted study	n11	n12	n21	n22	Odds ratio with 95% CI	p-value
Park et al 2017	314	7756	380	9620	0.94 [0.83, 1.06]	0.311
Phipps, Chlebowski, Prentice, et al 2011	147	72648	158	75933	0.97 [0.87, 1.08]	0.598
Gomes et al 2022	12	91	31	230	0.97 [0.88, 1.07]	0.563
Bigman et al 2022	0	7	121	879	0.97 [0.88, 1.07]	0.572
Wang et al 2022	307	63626	334	63718	1.01 [0.89, 1.14]	0.928

Supplementary Figure 16 Leave One Out Sensitivity Analysis for Breast Density

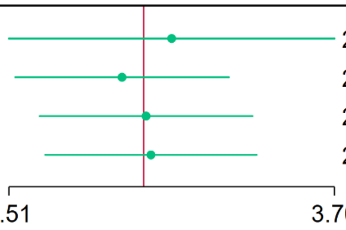

Omitted study	n11	n12	n21	n22	Odds ratio with 95% CI	p-value
Kleinstern et al 2021	79	1475	58	2264	2.36 [1.51, 3.70]	0.000
Ma et al 2009	20	36	17	99	2.06 [1.54, 2.76]	0.000
Shin et al 2018	21	311	10	310	2.20 [1.64, 2.95]	0.000
McCarthy et al 2021	17	15238	9	15143	2.23 [1.67, 2.98]	0.000

Supplementary Figure 17-a Leave One Out Sensitivity Analysis for BMI

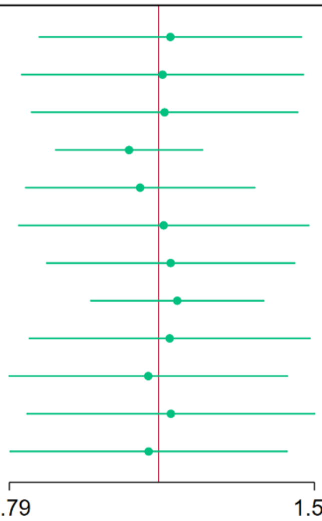

Omitted study	n11	n12	n21	n22	Odds ratio with 95% CI	p-value
Akinyemiju et al 2021	13	78	24	114	1.12 [0.85, 1.49]	0.417
Bigman et al 2022	44	324	31	240	1.11 [0.82, 1.50]	0.518
Gaudet et al 2011	7	118	62	981	1.11 [0.83, 1.48]	0.475
Gomes et al 2022	20	68	7	134	1.03 [0.88, 1.21]	0.730
Islam et al 2012	6	67	39	987	1.05 [0.82, 1.35]	0.681
Kleinstern et al 2021	64	1813	182	5393	1.11 [0.81, 1.52]	0.520
Klintman et al 2022	4	4148	26	16553	1.13 [0.86, 1.47]	0.388
Lee et al 2020	10	177	81	625	1.14 [0.95, 1.38]	0.165
Ma et al 2019	39	263	83	513	1.12 [0.83, 1.52]	0.454
Phipps, Chlebowski, Prentice, et al 2011	89	37207	66	37377	1.07 [0.79, 1.45]	0.651
Wang et al 2022	187	38725	228	43957	1.13 [0.82, 1.54]	0.455
McCarthy et al 2021	58	30074	54	38054	1.07 [0.80, 1.45]	0.645

Supplementary Figure 17-b Forest Plot for BMI after omitting Gomes et al 2022

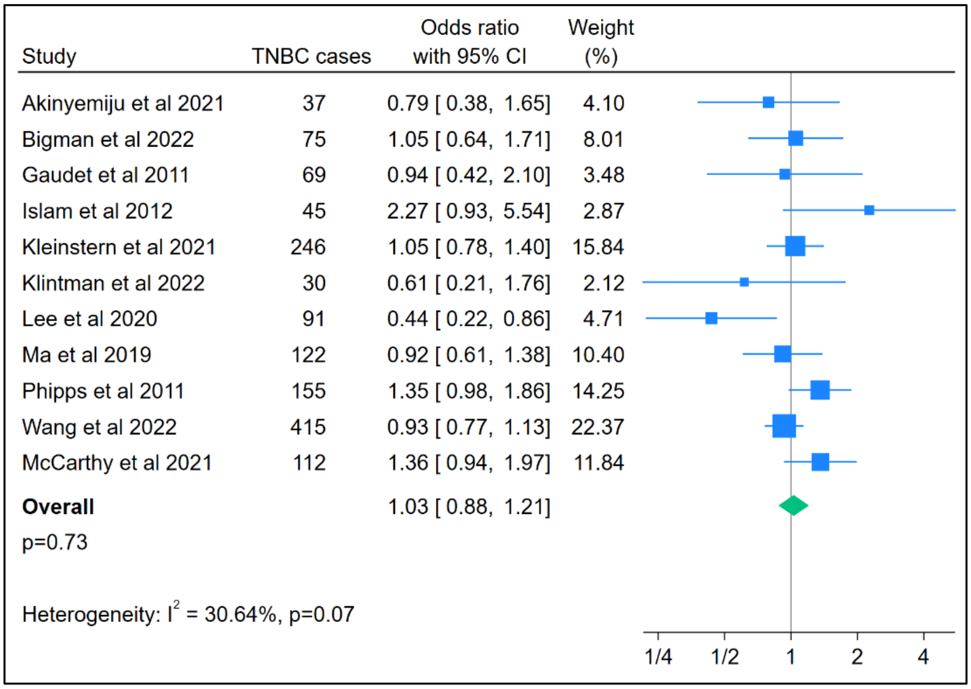

Supplementary Figure 18: Parity - Contour Funnel Plot & Egger's test Assessment

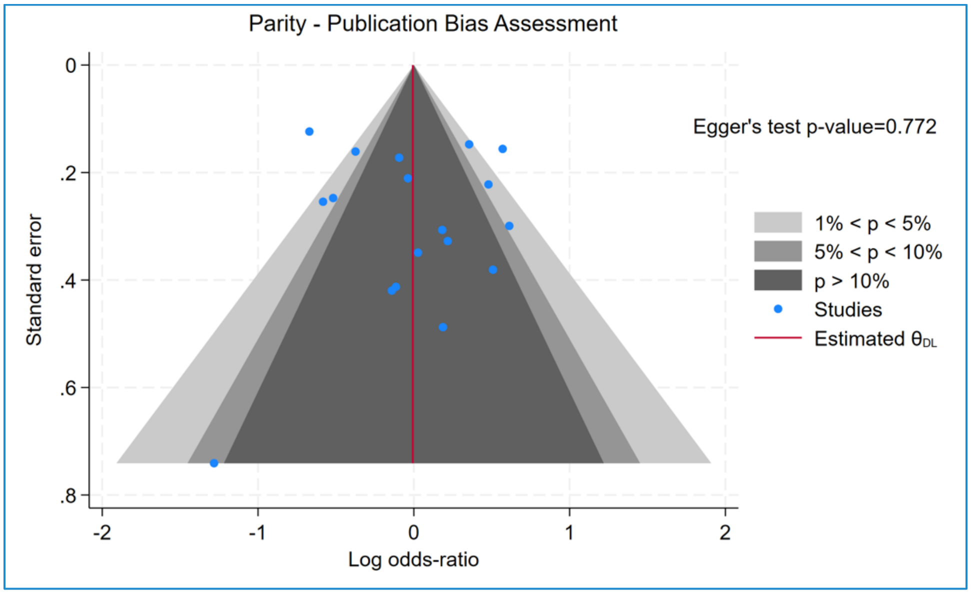

Supplementary Figure 19 –a: Breastfeeding - Contour Funnel Plot and Egger's Test Assessment

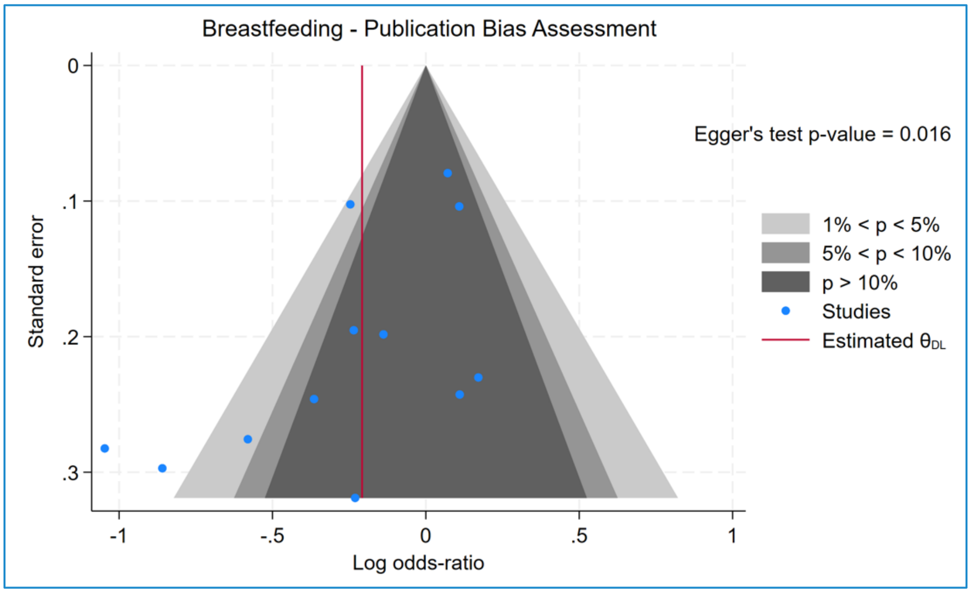

Supplementary Figure 19-b: Breastfeeding - Trim and Fill Plot

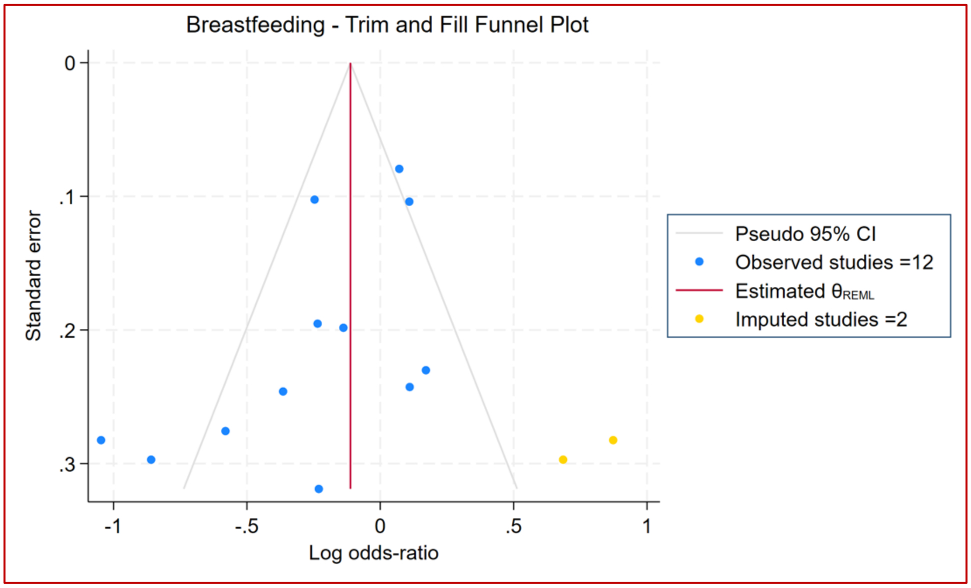

Supplementary Figure 20: Duration of Breastfeeding - Contour Funnel Plot & Egger's test Assessment

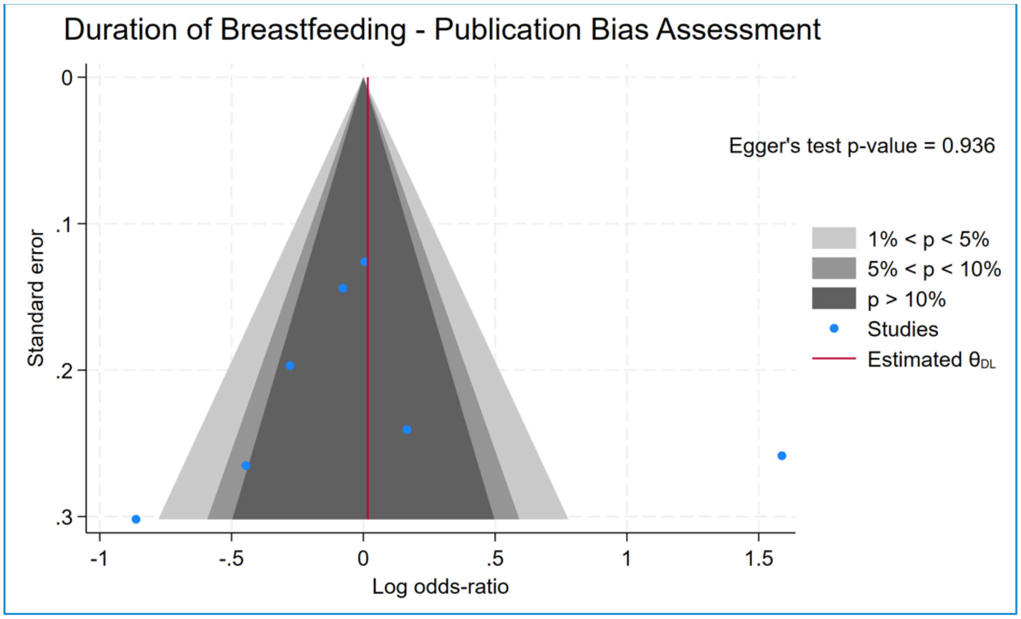

Supplementary Figure 21: Age at Menarche - Contour Funnel Plot & Egger's test Assessment

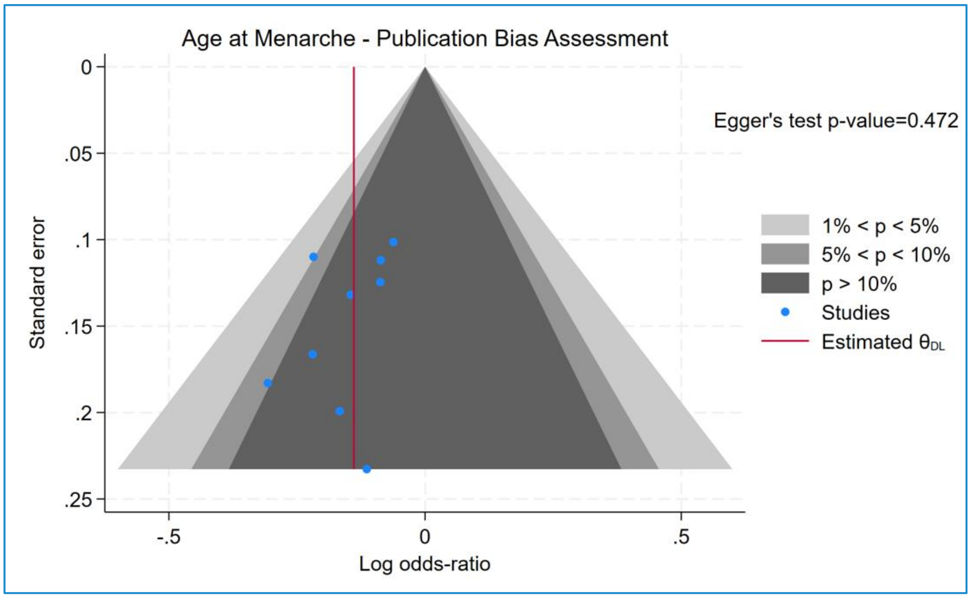

Supplementary Figure 22: Age at 1st Live Birth - Contour Funnel Plot & Egger's test Assessment

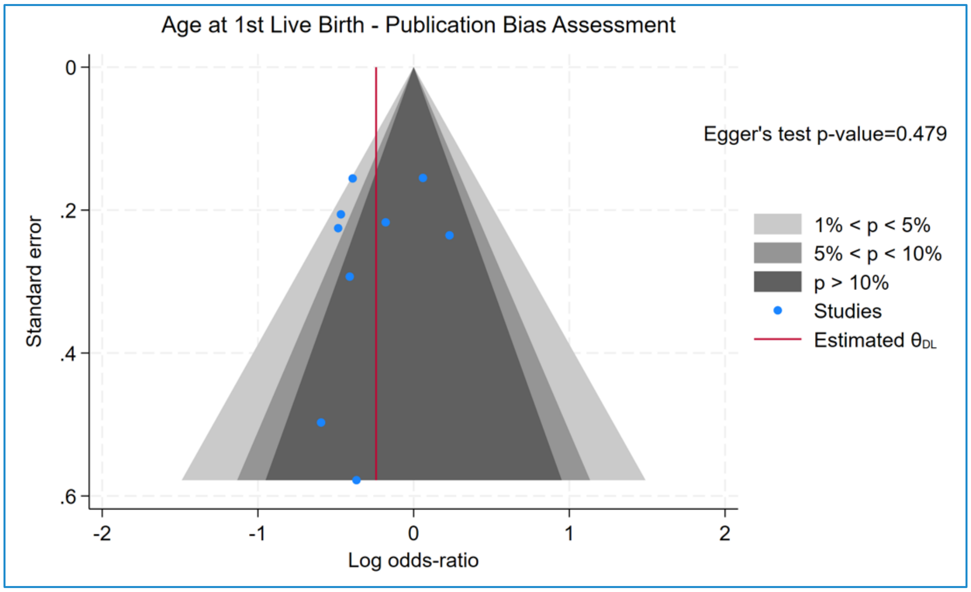

Supplementary Figure 23: OC Use - Contour Funnel Plot & Egger's test Assessment

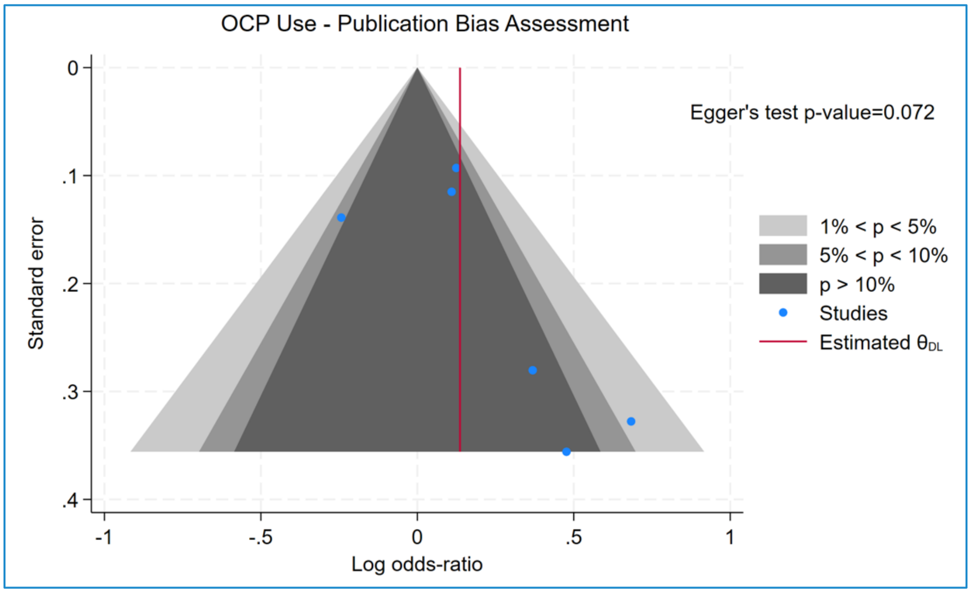

Supplementary Figure 24: Duration of OC Use - Contour Funnel Plot & Egger's test Assessment

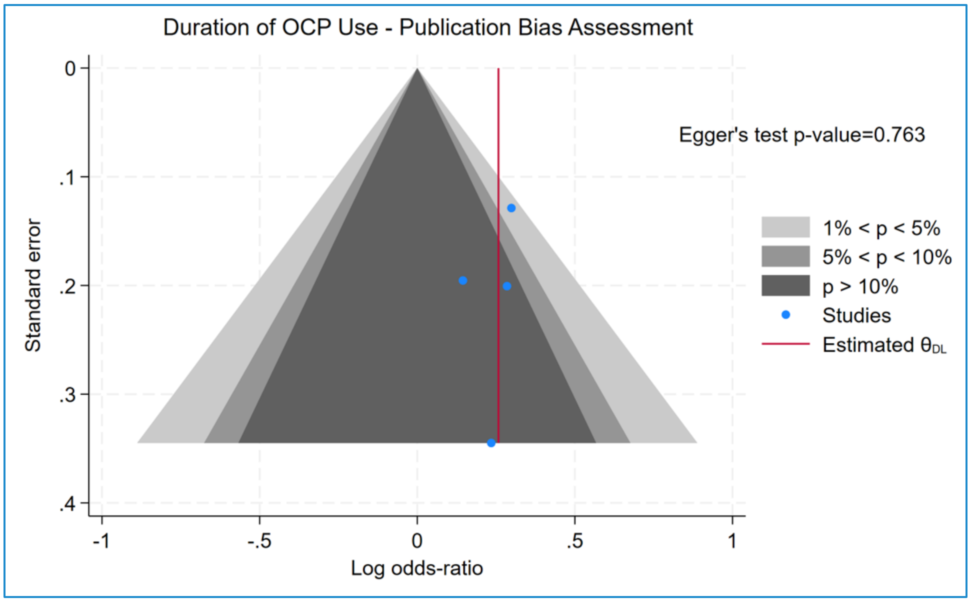

Supplementary Figure 25-a: MHT Use - Contour Funnel Plot & Egger's test Assessment

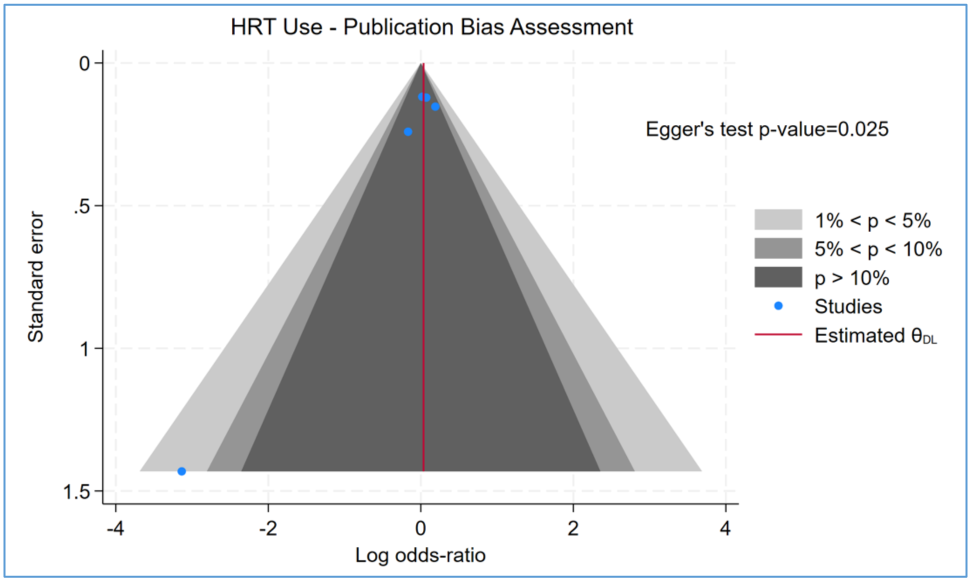

Supplementary Figure 25-b: MHT Use – Trim and Fill Plot

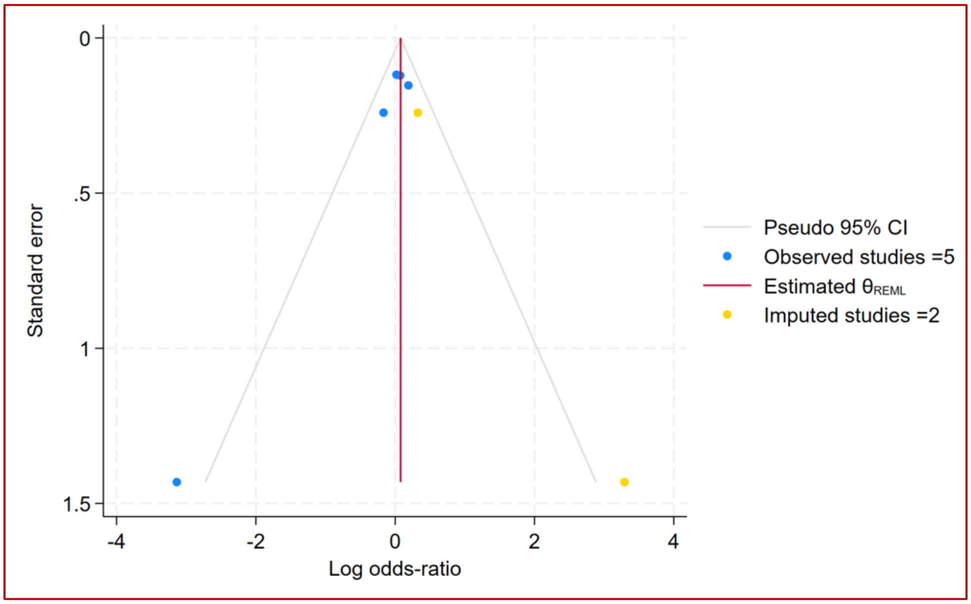

Supplementary Figure 26: Family History - Contour Funnel Plot & Egger's test Assessment

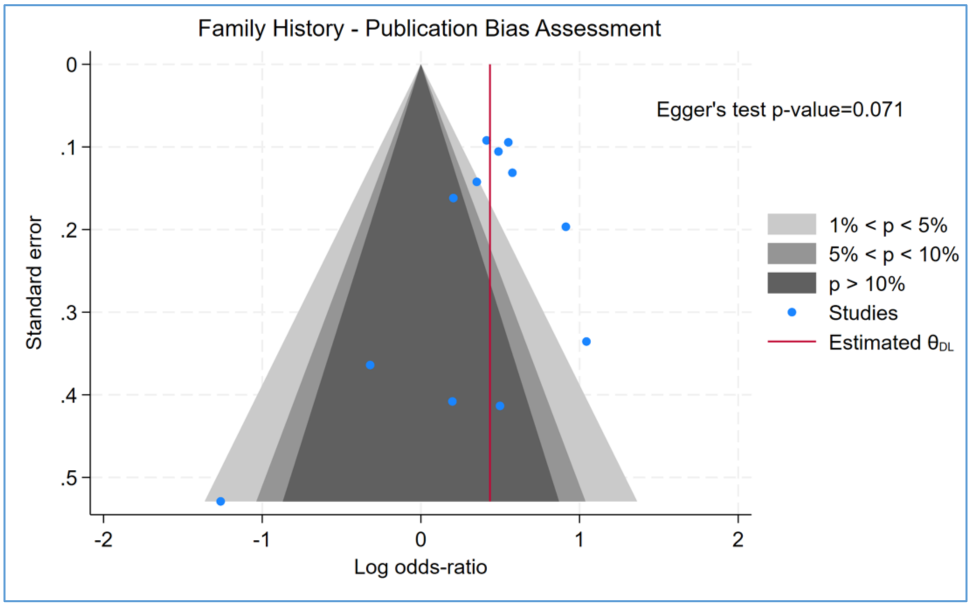

Supplementary Figure 27: Alcohol - Contour Funnel Plot & Egger's test Assessment

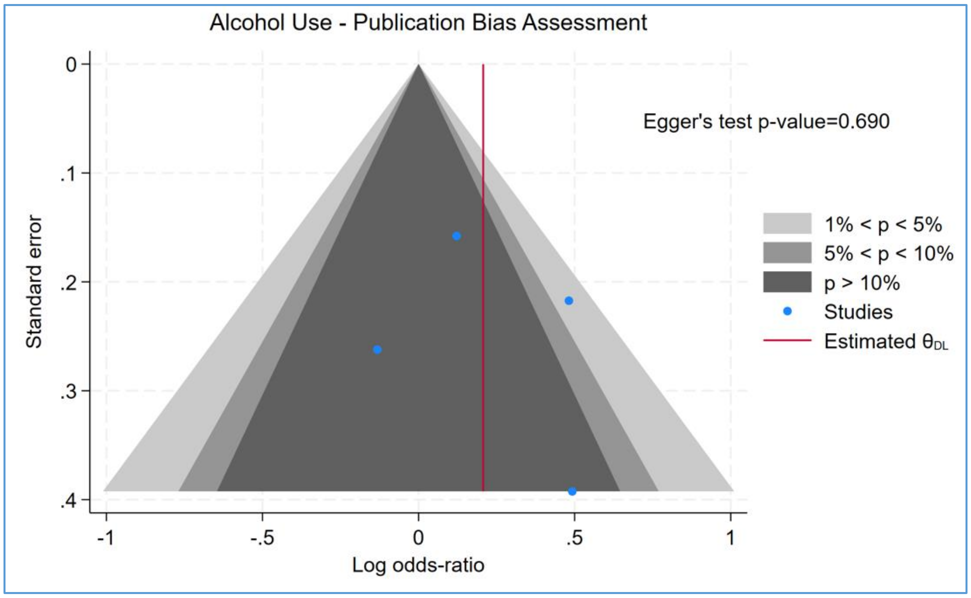

Supplementary Figure 28: Smoking - Contour Funnel Plot & Egger's test Assessment

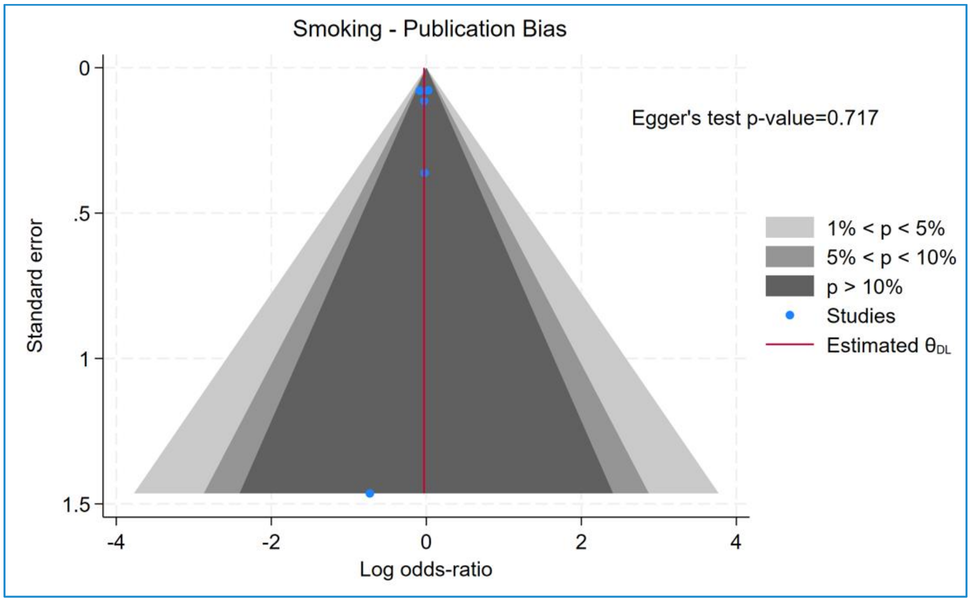

Supplementary Figure 29: BMI - Contour Funnel Plot & Egger's test Assessment

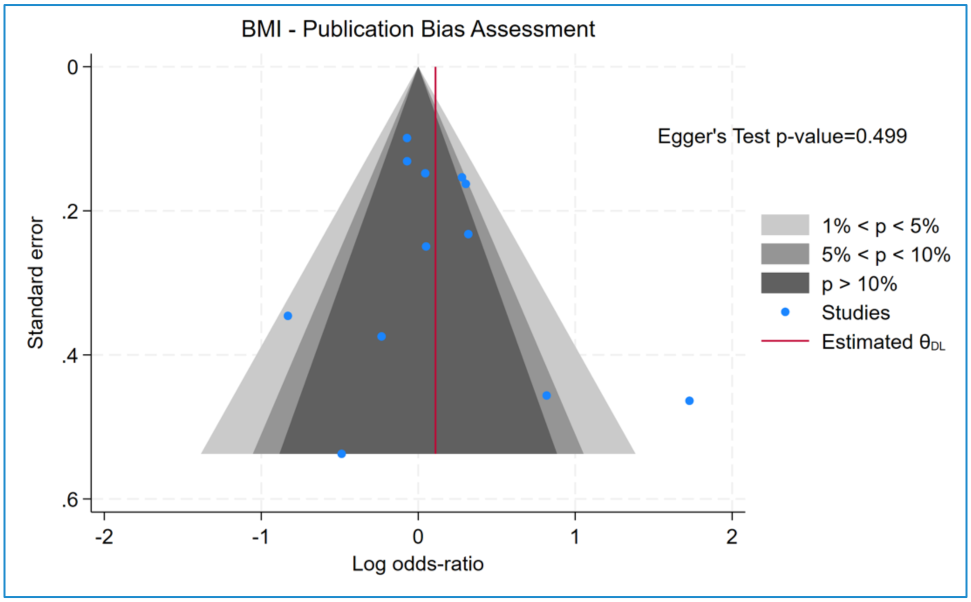

Supplementary Figure 30: Breast Density - Contour Funnel Plot & Egger's test Assessment

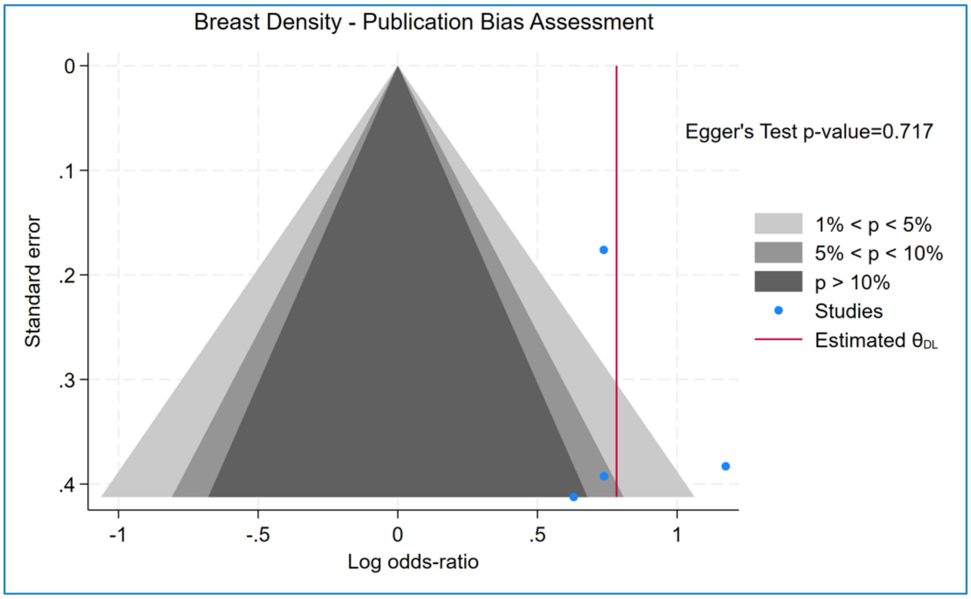

Supplementary Table 1: PRISMA Checklist

#	Item	Guidance	On page #	Author Comments
**Title**				
1	Title	Identify the report as a systematic review, or systematic review and meta-analysis, as appropriate.	Title page	
**Abstract**				
2	Structured summary	Provide a structured summary including, as applicable:<br>• Background;<br>• Objectives;<br>• Data sources;<br>• Study eligibility criteria, participants, and interventions;<br>• Study appraisal and synthesis methods;<br>• Results;<br>• Limitations; conclusions and implications of key findings;<br>• Systematic review registration number.	2	
**Introduction**				
3	Rationale	Describe the rationale for the review in the context of what is already known.	3	
4	Objectives	Provide an explicit Population-Intervention-Comparator-Outcome-Study Design (PICOS) or Population-Exposure-Comparator-Outcome-Study Design (PECOS) statement as appropriate, detailing the following in relation to the research questions being asked:<br>• Participants<br>• Interventions / Exposures (as appropriate)<br>• Comparisons<br>• Outcomes<br>• Study design	3	
**Methods**				
5	Protocol and registration	Indicate if a review protocol exists, if and where it can be accessed (e.g. web address), and registration information including registration number (if available).	3	Protocol Regissstration number: PROSPERO 2021 CRD42021254594
6	Eligibility criteria	Specify study characteristics (e.g. PICOS/PECOS, length of exposure) and report characteristics (e.g. years considered, language, publication status) used as criteria for eligibility, giving rationale.	4	

7	Informatio n sources	Describe all information sources (e.g. databases with dates of coverage, contact with study authors to identify additional studies) in the search, and date last searched.	3	
8	Search	Present full electronic search strategy for at least one database, including any limits used, such that it could be repeated.	3	
9	Study selection	State the process for selecting studies (i.e., screening, eligibility, included in systematic review, and, if applicable, included in the meta-analysis).	4	
10	Data collection process	Describe method of data extraction from reports (e.g., piloted forms, independently, in duplicate) and any processes for obtaining and confirming data from investigators.	5	
11	Data items	List and define all variables for which data were sought (e.g., PICOS/PECOS, funding sources) and any assumptions and simplifications made.	5	
12	Risk of bias in individual studies	Describe methods used for assessing risk of bias of individual studies (including specification of whether this was done at the study or outcome level), and how this information is to be used in any data synthesis.	5	
13	Summary measures	State the principal summary measures (e.g., risk ratio, difference in means).	5	
14	Synthesis of results	Describe the methods of handling data and combining results of studies, if done, including measures of consistency (e.g., I^2) for each meta-analysis.	5	
15	Risk of bias across studies	Specify any assessment of risk of bias that may affect the cumulative evidence (e.g., publication bias, selective reporting within studies).	5	
16	Additional analyses	Describe methods of additional analyses (e.g., sensitivity or subgroup analyses, meta-regression), if done, indicating which were pre-specified.	5	
**Results**				
17	Study selection	Give numbers of studies screened, assessed for eligibility, and included in the review, with reasons for exclusions at each stage, illustrated with a PRISMA flow diagram.	6	
18	Study characteris tics	For each study, present in a summary table the characteristics for which data were extracted (e.g., study size, PICOS/PECOS, follow-up period) and provide the citations.	6	
19	Risk of bias within studies	Present data on risk of bias of each study and, if available, any outcome level assessment (see item 12).	6	
20	Results of individual studies	For all outcomes considered (benefits or harms), present, for each study: (a) simple summary data for each intervention group (b) effect estimates and confidence intervals, ideally with a forest plot (unless such a plot would be misleading)	6 to 8	
21	Synthesis of results	Present results of each meta-analysis done, including confidence intervals and measures of consistency.	6 to 8	
22	Risk of bias across studies	Present results of any assessment of risk of bias across studies (see Item 15).	12	

23	Additional analysis	Give results of additional analyses, if done (e.g., sensitivity or subgroup analyses, meta-regression [see Item 16]).	6 to 8	
24	Summary of evidence	Summarize the main findings including the strength of evidence for each main outcome; consider their relevance to key groups (e.g., researchers, users, and policy makers).	9	
25	Limitations	Discuss limitations at study and outcome level (e.g., risk of bias), and at review-level (e.g., incomplete retrieval of identified research, reporting bias).	10 and 11	
26	Conclusion s	Provide a general interpretation of the results in the context of other evidence, and implications for future research.	11	
27	Funding	Describe sources of funding for the systematic review and other support (e.g., supply of data); role of funders for the systematic review.	18	

Supplementary Figure 31: Preferred Reporting Items for Systematic Reviews and Meta Analyses (PRISMA) Indicating Identification and Selection of Studies.

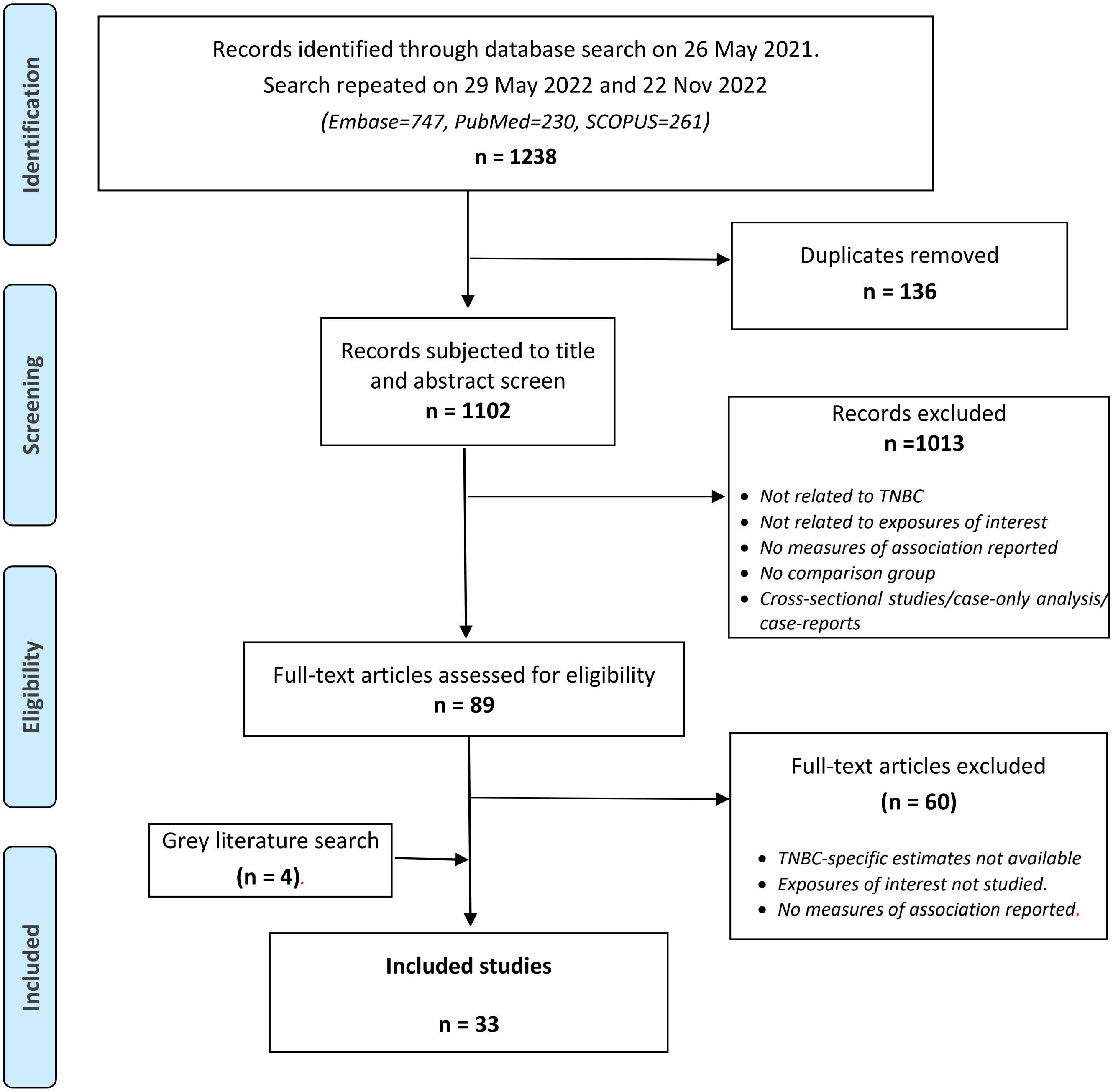

Supplementary Figure 32: Odds of TNBC in those with >=12 months of breastfeeding vs. 0 months.

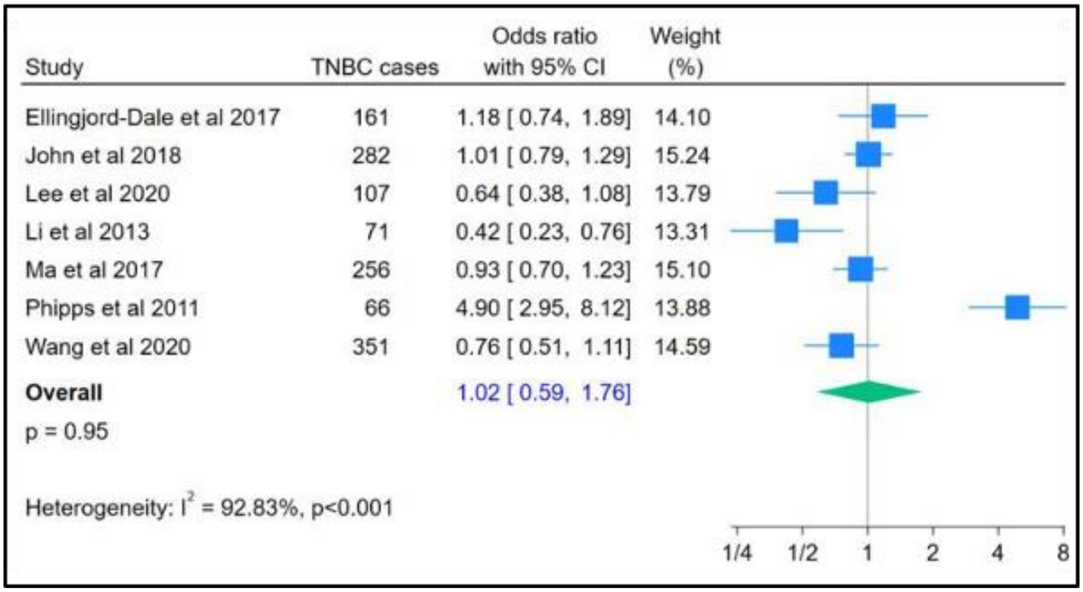

Supplementary Figure 33: Odds of TNBC in those who ever used OC vs. those who never did.

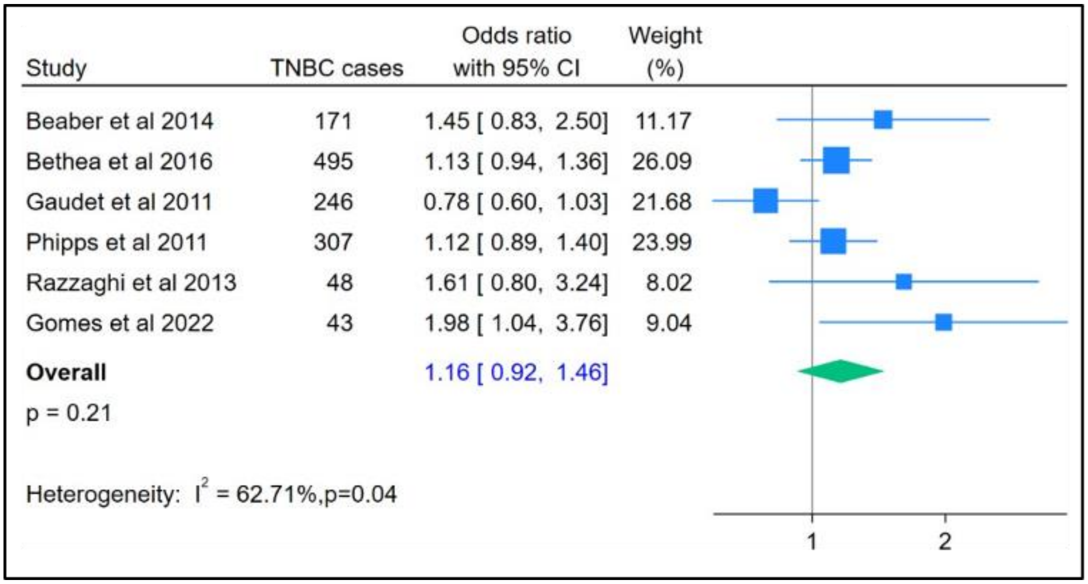

Supplement: djae056_Supplementary_Data [file djae056_supplementary_data.pdf]
